# Supplementary material for: Site-Specific Conversion of Cysteine in a Protein to Dehydroalanine Using 2-Nitro-5-thiocyanatobenzoic Acid
Source: Molecules. 2021 Apr 29;26(9):2619. doi: 10.3390/molecules26092619 (PMC8125731; doi:10.3390/molecules26092619)
Supplement: Supplementary file 1 [file molecules-26-02619-s001.zip › molecules-1195052-supplementary.pdf]

Supporting Information

Site-Specific Conversion of Cysteine in a Protein to Dehydroalanine using 2-Nitro-5-thiocyanatobenzoic acid

Yuchen Qiao[1], Ge Yu[1], and Wenshe Ray Liu [1,2,3,4]*

[1] The Texas A&M Drug Discovery Laboratory, Department of Chemistry, Texas A&M University, College Station, TX 77843, USA
[2] Department of Biochemistry & Biophysics, Texas A&M University, College Station, TX 77843, USA
[3] Molecular & Cellular Medicine Department, College of Medicine, Texas A&M University, College Station, TX 77843, USA
[4] Institute of Biosciences and Technology and Department of Translational Medical Sciences, College of Medicine, Texas A&M University, Houston, TX 77030, USA

* Author to whom correspondence should be addressed.

SI Table of Contents **Page**

Contents	Page
Supplementary Figures S1-S46<br>(SDS-PAGE analysis, ESI-MS analysis)	S3
Supplementary Tables S1<br>(Amino acid sequences of recombinant proteins)	S46
Supplementary Tables S1<br>(DNA sequences of recombinant proteins)	S48

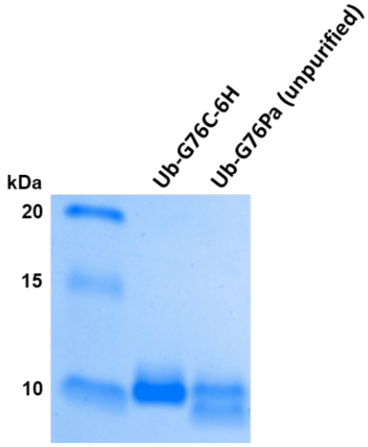

Figure S1. SDS-PAGE analysis of original Ub-G76C-6H (left line beside protein ladder) and ACPL reaction product mixture (right line) between Ub-G76C-6H and Pa without further Ni purification. The product line indicated the combination of two product where the bottom band referred to ligation product, Ub-G76Pa.

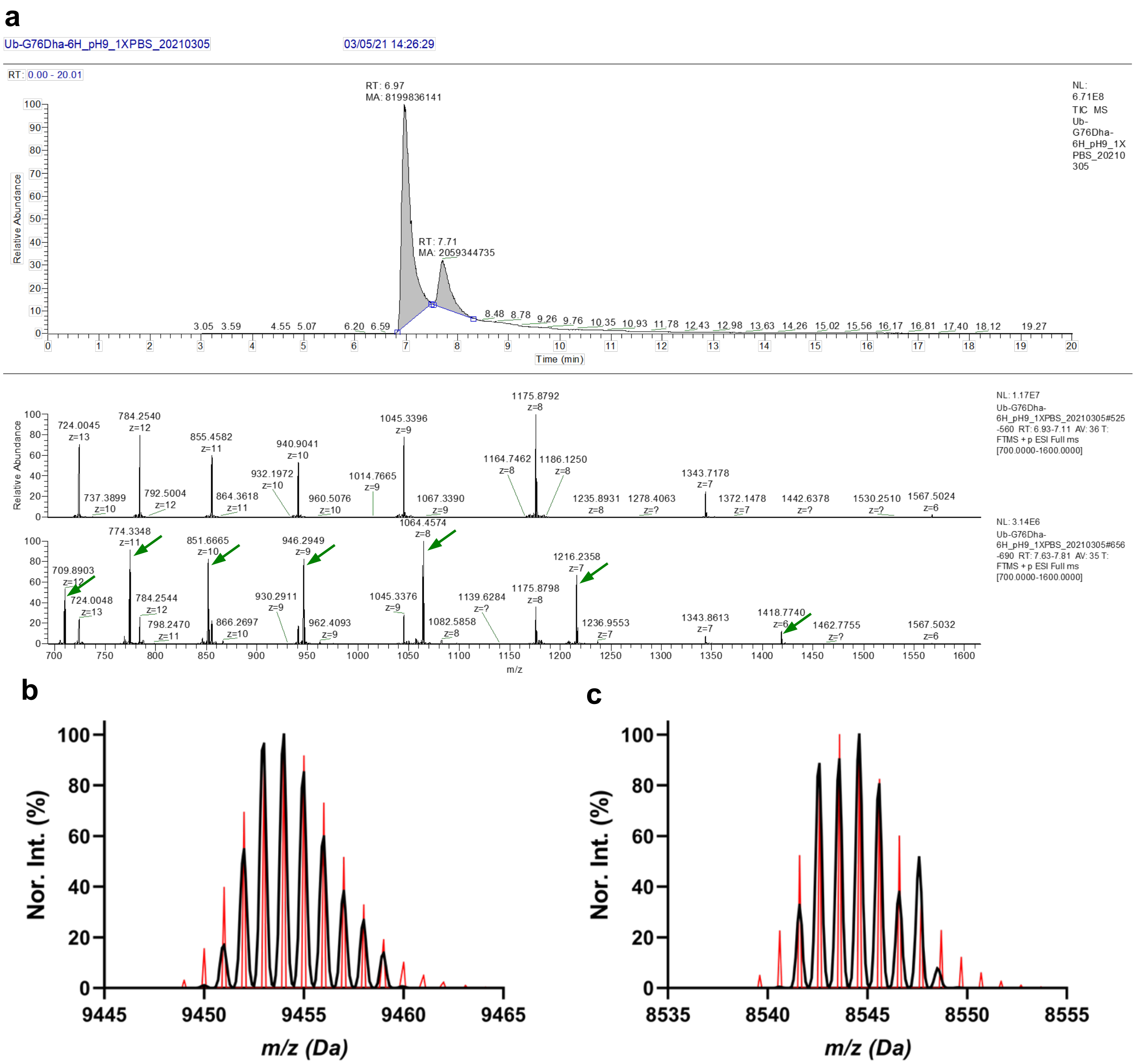

Figure S2. ESI-LC-MS analysis of product mixture of NTCB induced Dha formation reaction using Ub-G76C-6H as the substrate. Reaction condition: 1× PBS pH 9, 37 °C, 18 h incubation. (**a**) The top mass diagram that came from the 6.97 min peak represented Ub-G76Dha-6H. The major mass signal pattern indicated in green in the bottom mass diagram that came from the 7.71 min peak designated the formation of hydrolysis product, Ub$_{(1\text{-}75)}$. (**b**) Deconvoluted mass spectrum (black line) of the Dha derivative product formed via Michael addition reaction between Pa and Ub-G76Dha-6H, and the theoretic natural abundance of its monoisotopic peaks (red line). (**c**) Deconvoluted mass spectrum (black line) of the Ub-G76Pa, and the theoretic natural abundance of its monoisotopic peaks (red line).

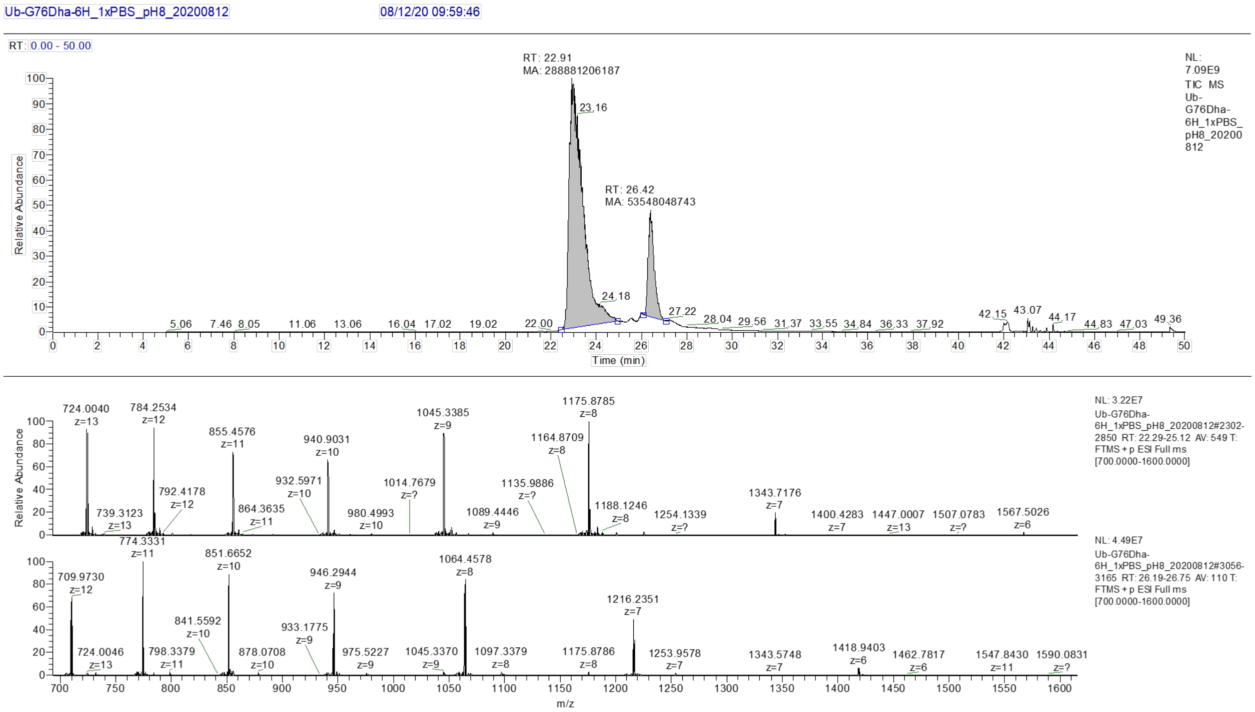

Figure S3. ESI-LC-MS analysis of product mixture of NTCB induced Dha formation reaction using Ub-G76C-6H as the substrate. Reaction condition: 1× PBS pH 8, 37 °C, 18 h incubation. The mass signal pattern in the top mass diagram that came from the 22.91 min peak represented Ub-G76Dha-6H. The major mass signal pattern indicated in green in the bottom mass diagram that came from the 26.42 min peak designated the formation of hydrolysis product, $Ub_{(1-75)}$.

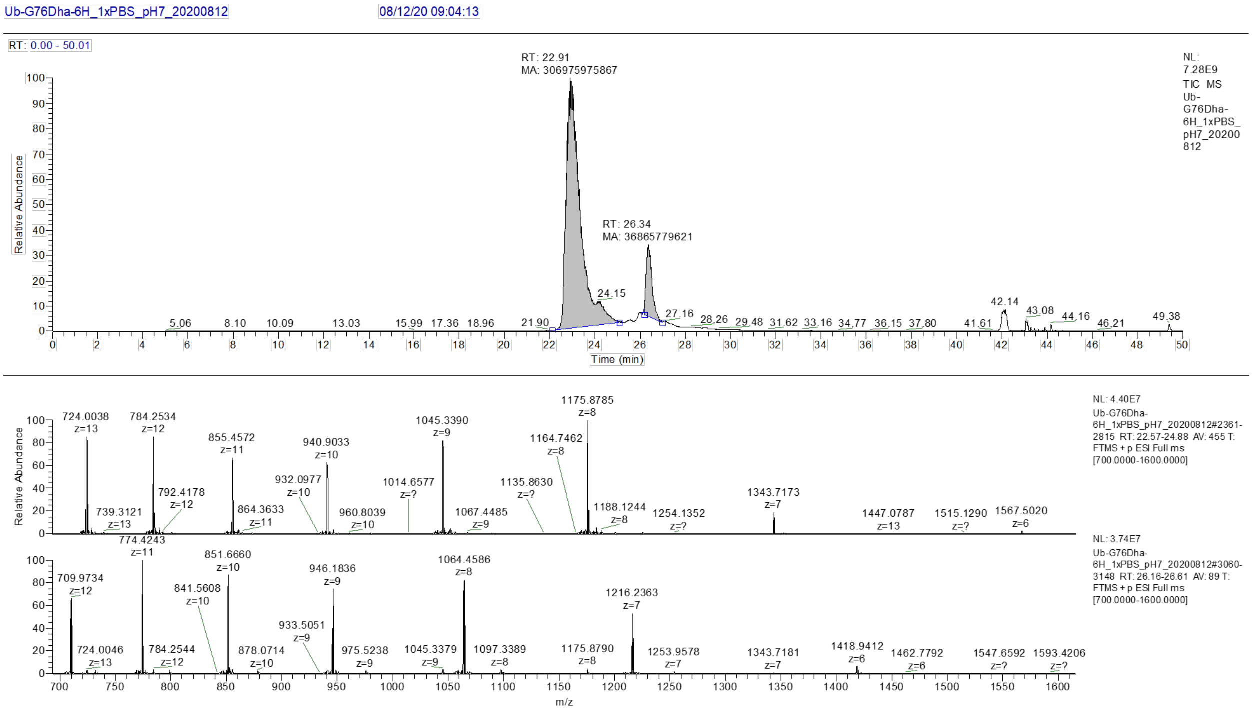

Figure S4. ESI-LC-MS analysis of product mixture of NTCB induced Dha formation reaction using Ub-G76C-6H as the substrate. Reaction condition: 1× PBS pH 7, 37 °C, 18 h incubation. The mass signal pattern in the top mass diagram that came from the 22.91 min peak represented Ub-G76Dha-6H. The bottom mass diagram that came from the 26.34 min peak designated the formation of hydrolysis product, $Ub_{(1\text{-}75)}$.

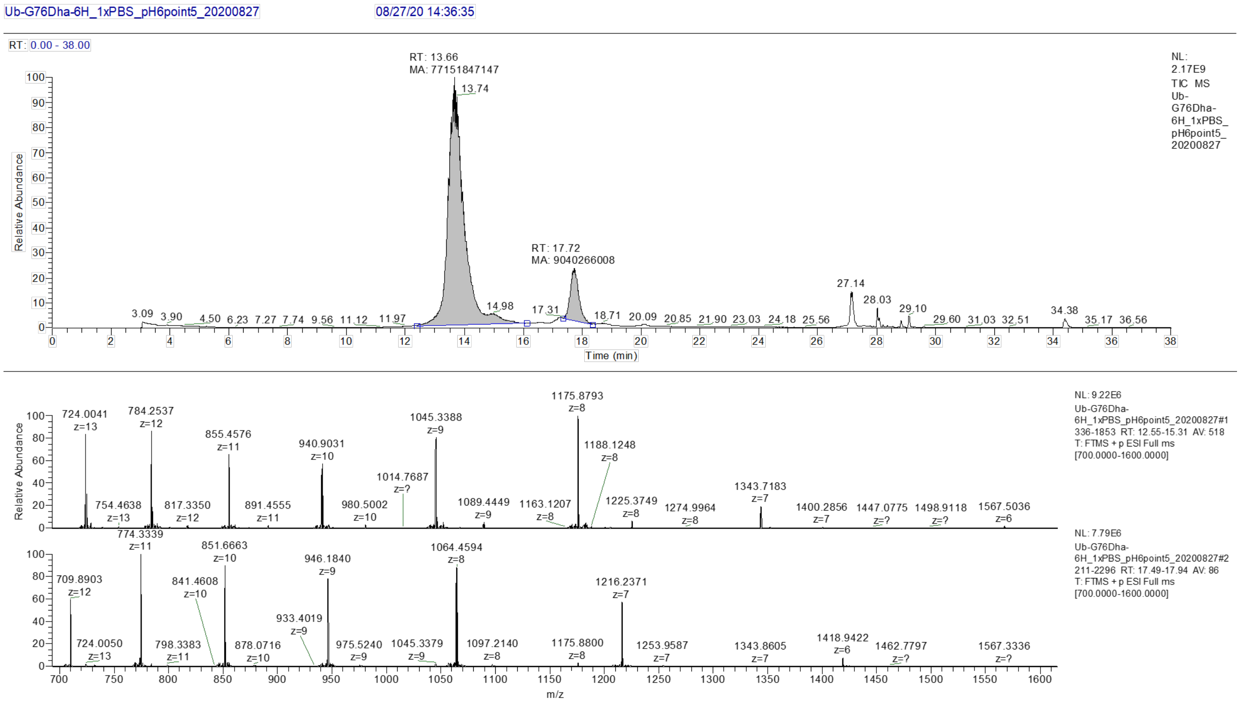

Figure S5. ESI-LC-MS analysis of product mixture of NTCB induced Dha formation reaction using Ub-G76C-6H as the substrate. Reaction condition: 1× PBS pH 6.5, 37 °C, 18 h incubation. The mass signal pattern in the top mass diagram that came from the 13.66 min peak represented Ub-G76Dha-6H. The bottom mass diagram that came from the 17.72 min peak designated the formation of hydrolysis product, $Ub_{(1-75)}$.

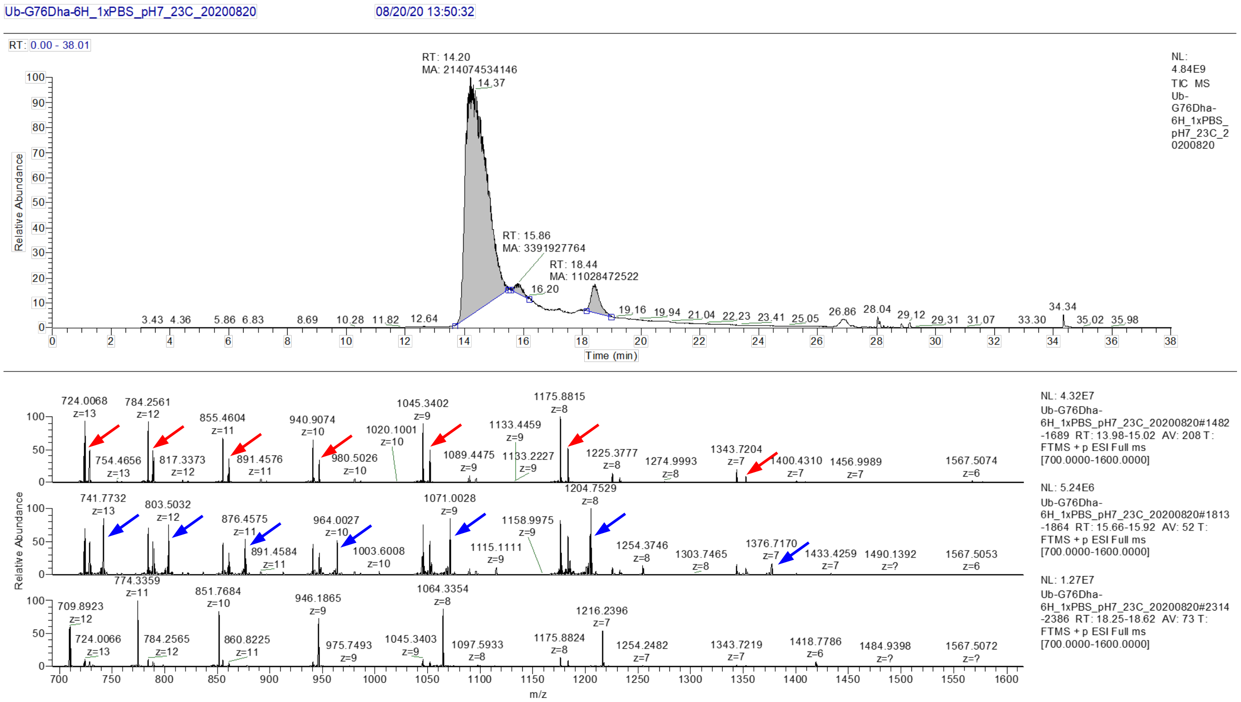

Figure S6. ESI-LC-MS analysis of product mixture of NTCB induced Dha formation reaction using Ub-G76C-6H as the substrate. Reaction condition: 1× PBS pH 7, 23 °C, 18 h incubation. The major mass signal pattern in the top mass diagram that came from the 14.20 min peak represented Ub-G76Dha-6H. The minor mass signal pattern indicated in red on the right side of each Dha product signal showed the formation of the +CN product, Ub-G76C(CN)-6H. The mass signal pattern indicated in blue in the middle mass diagram that came from the 15.86 min peak determined the formation of +TNB product, Ub-G76C(TNB)-6H. The bottom mass diagram that came from the 18.44 min peak designated the formation of hydrolysis product, $Ub_{(1\text{-}75)}$.

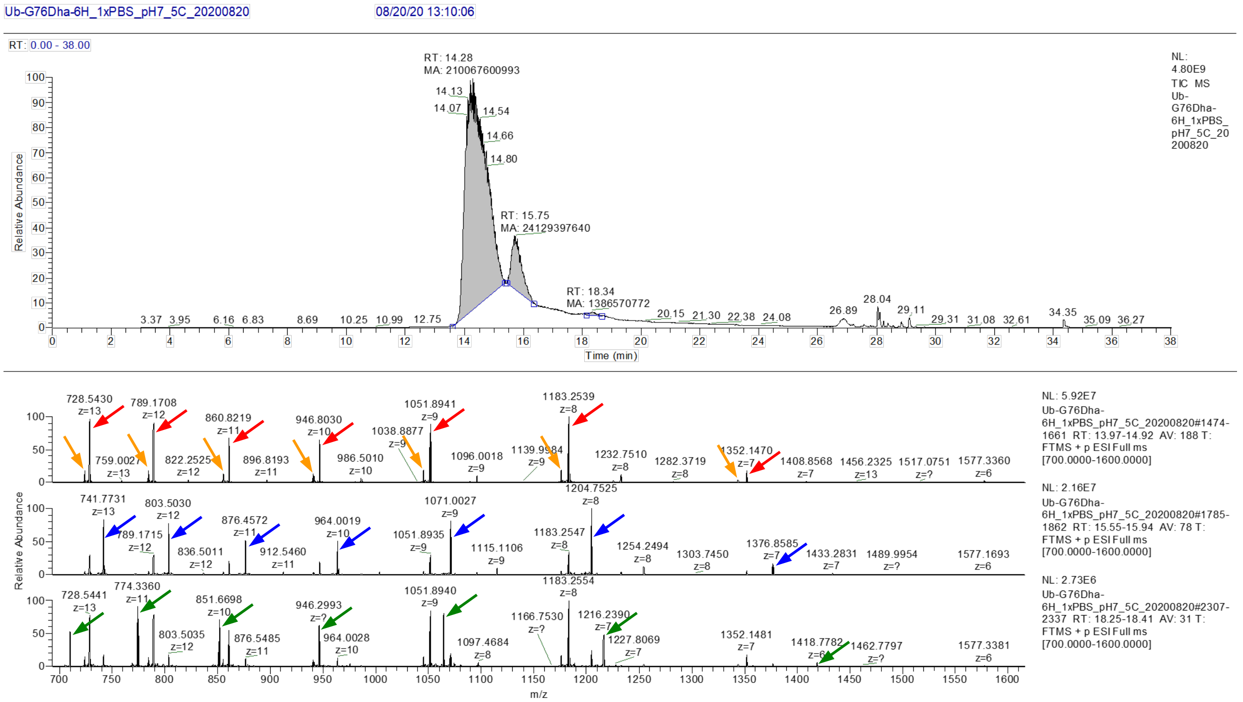

Figure S7. ESI-LC-MS analysis of product mixture of NTCB induced Dha formation reaction using Ub-G76C-6H as the substrate. Reaction condition: 1× PBS pH 7, 5 °C, 18 h incubation. The major mass signal pattern indicated in red in the top mass diagram that came from the 14.28 min peak showed the formation of +CN product, Ub-G76C(CN)-6H. The minor mass signal pattern indicated in orange on the left side of each +CN product signal represented Ub-G76Dha-6H. The mass signal pattern indicated in blue in the middle mass diagram that came from the 15.75 min peak determined the formation of +TNB product, Ub-G76C(TNB)-6H. The mass signal pattern indicated in green in the bottom mass diagram that came from the 18.34 min peak designated the formation of hydrolysis product, $Ub_{(1-75)}$.

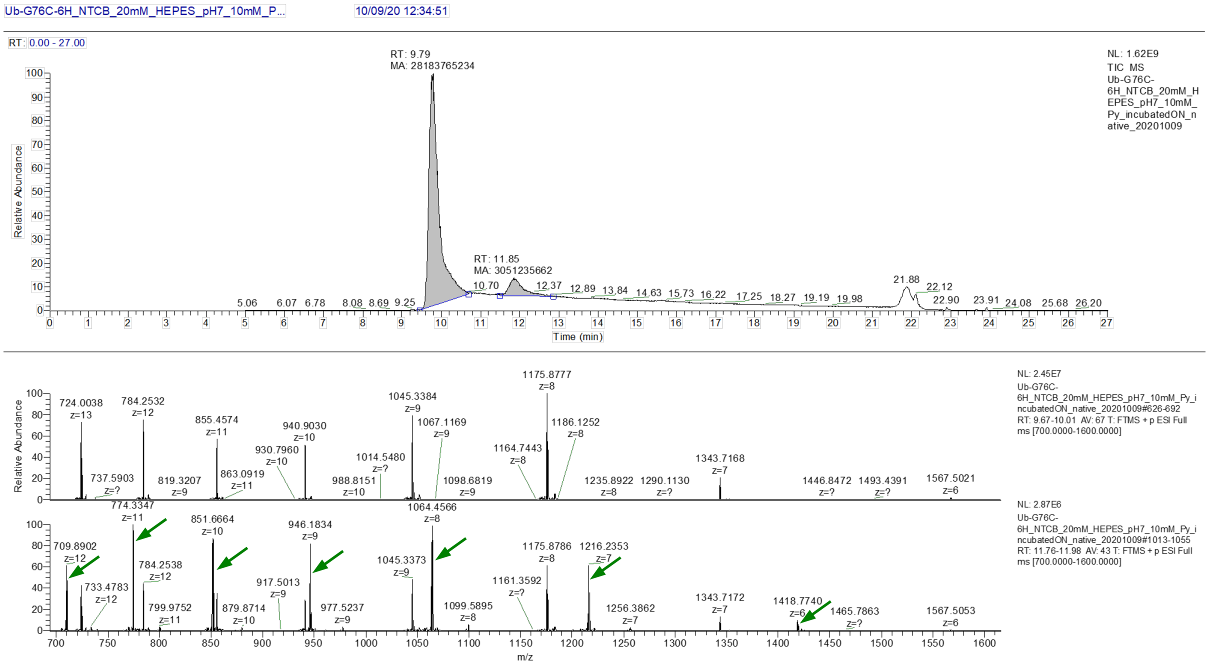

Figure S8. ESI-LC-MS analysis of product mixture of NTCB induced Dha formation reaction using Ub-G76C-6H as the substrate. Reaction condition: 20 mM HEPES pH 7, 37 °C, 18 h incubation. The top mass diagram that came from the 9.79 min peak represented Ub-G76Dha-6H. The mass signal pattern indicated in green in the bottom mass diagram that came from the 11.85 min peak designated the formation of hydrolysis product, $Ub_{(1-75)}$.

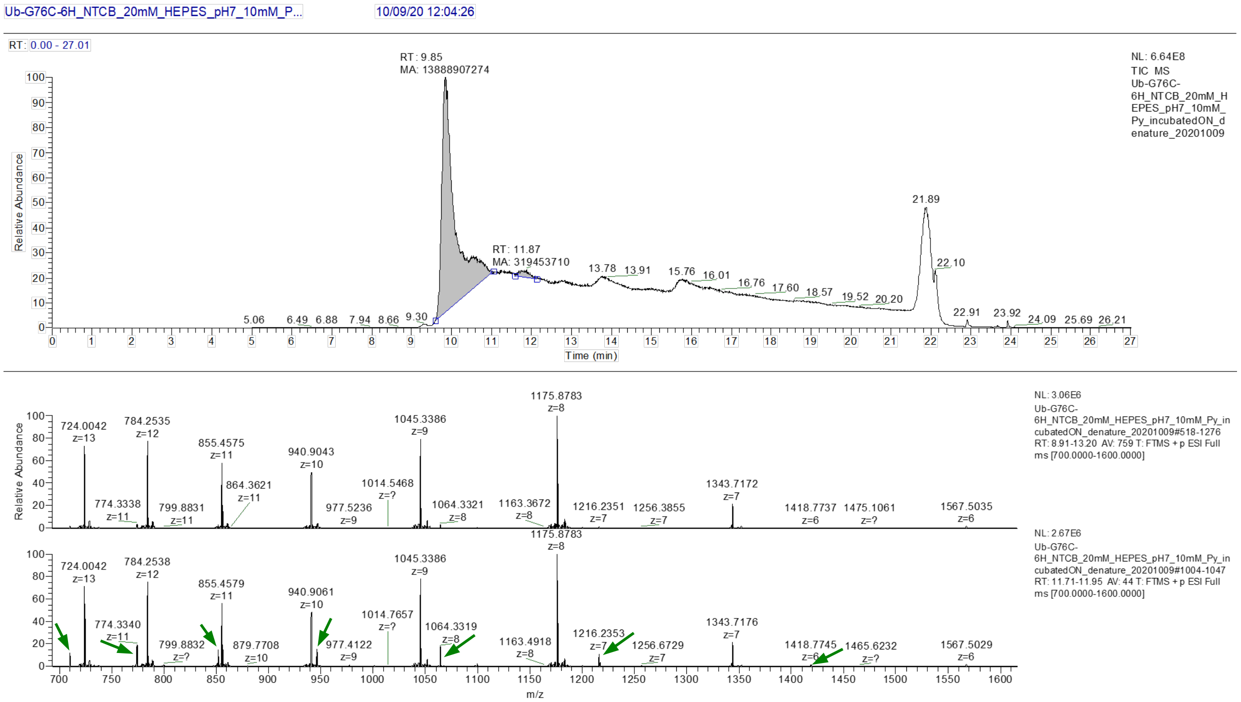

Figure S9. ESI-LC-MS analysis of product mixture of NTCB induced Dha formation reaction using Ub-G76C-6H as the substrate. Reaction condition: 20 mM HEPES pH 7, 6 M GndCl, 37 °C, 18 h incubation. The top mass diagram that came from the 9.85 min peak represented Ub-G76Dha-6H. The mass signal pattern indicated in green in the bottom mass diagram that came from the 11.87 min peak designated the formation of hydrolysis product, $Ub_{(1\text{-}75)}$.

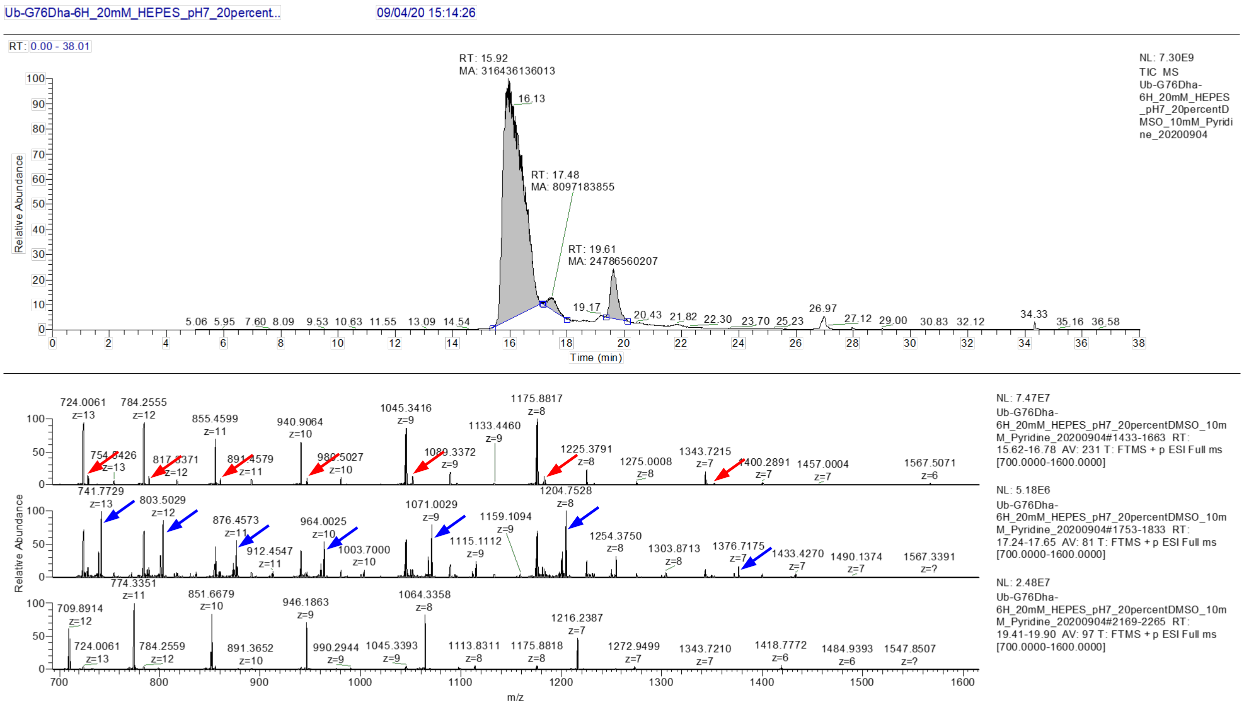

Figure S10. ESI-LC-MS analysis of product mixture of NTCB induced Dha formation reaction using Ub-G76C-6H as the substrate. Reaction condition: 20 mM HEPES pH 7, 20% DMSO, 37 °C, 18 h incubation. The major mass signal pattern in the top mass diagram that came from the 15.92 min peak represented Ub-G76Dha-6H. The minor mass signal pattern indicated in red on the right side of each Dha product signal showed the formation of +CN product, Ub-G76C(CN)-6H. The mass signal pattern indicated in blue in the middle mass diagram that came from the 17.48 min peak determined the formation of +TNB product, Ub-G76C(TNB)-6H. The mass signal pattern in the bottom mass diagram that came from the 19.61 min peak designated the formation of hydrolysis product, $Ub_{(1-75)}$.

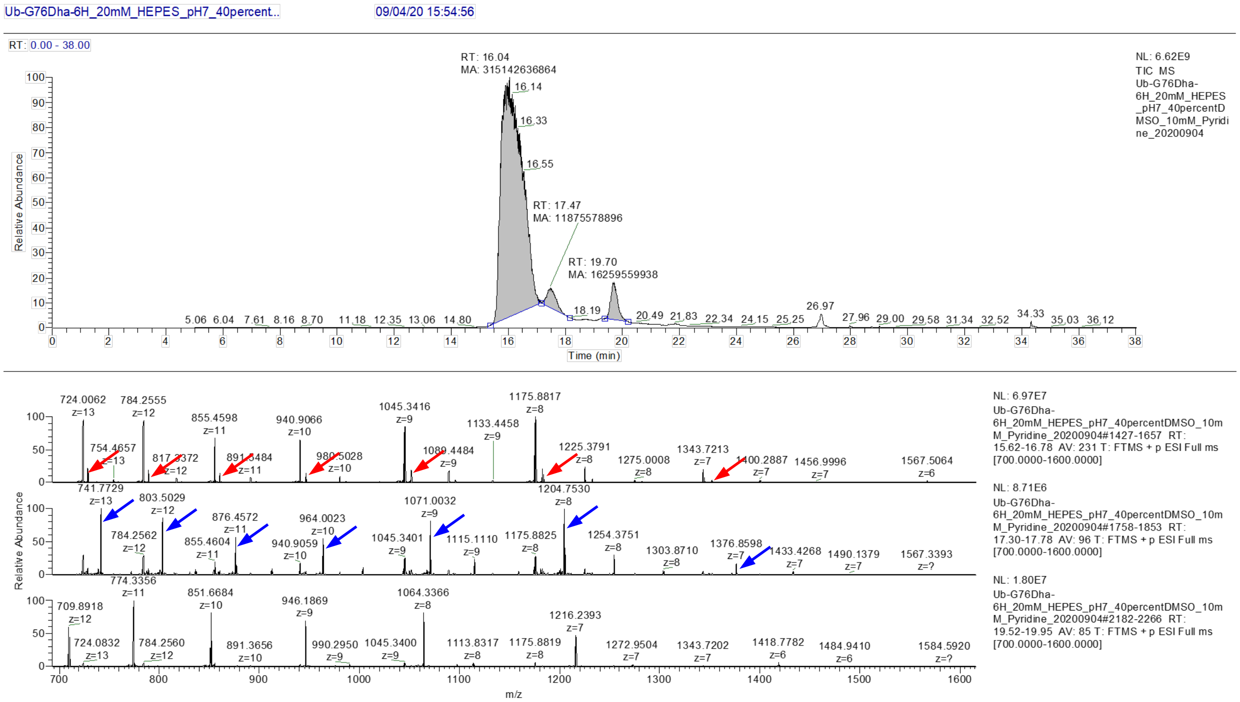

Figure S11. ESI-LC-MS analysis of product mixture of NTCB induced Dha formation reaction using Ub-G76C-6H as the substrate. Reaction condition: 20 mM HEPES pH 7, 40% DMSO, 37 °C, 18 h incubation. The major mass signal pattern in the top mass diagram that came from the 16.04 min peak represented Ub-G76Dha-6H. The minor mass signal pattern indicated in red on the right side of each Dha product signal showed the formation of +CN product, Ub-G76C(CN)-6H. The mass signal pattern indicated in blue in the middle mass diagram that came from the 17.47 min peak determined the formation of +TNB product, Ub-G76C(TNB)-6H. The mass signal pattern in the bottom mass diagram that came from the 19.70 min peak designated the formation of hydrolysis product, $Ub_{(1-75)}$.

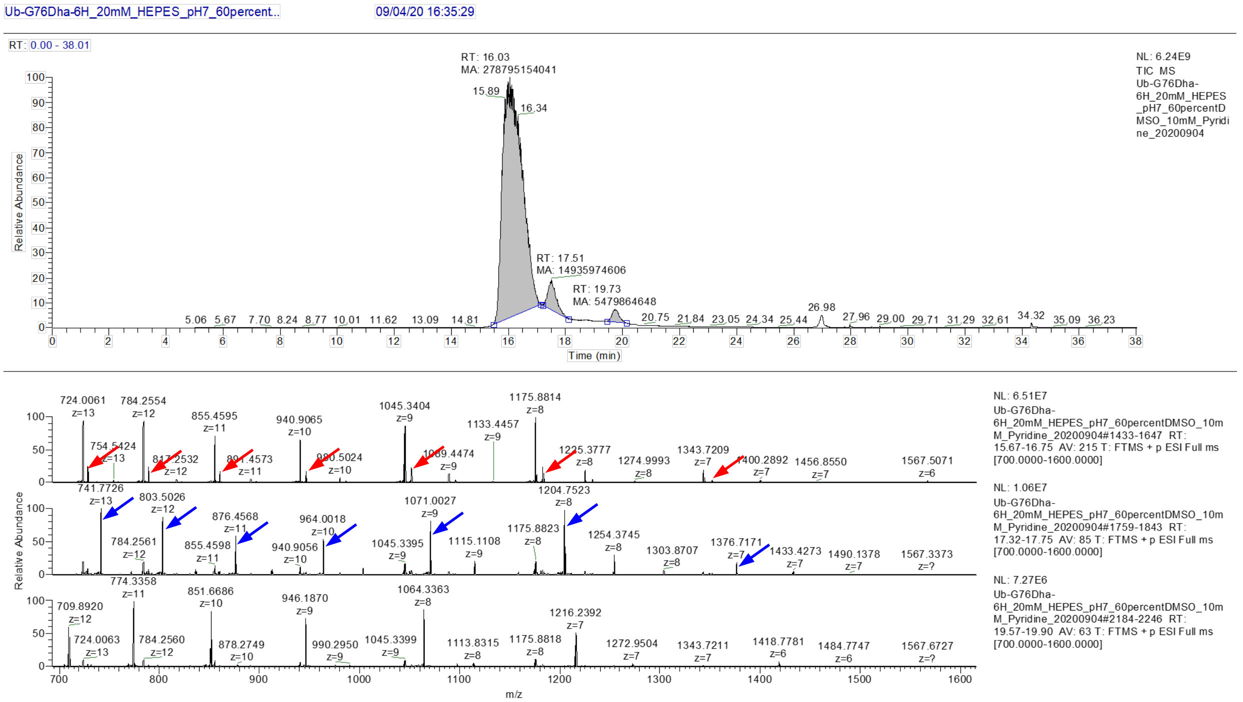

Figure S12. ESI-LC-MS analysis of product mixture of NTCB induced Dha formation reaction using Ub-G76C-6H as the substrate. Reaction condition: 20 mM HEPES pH 7, 60% DMSO, 37 °C, 18 h incubation. The major mass signal pattern in the top mass diagram that came from the 16.03 min peak represented Ub-G76Dha-6H. The minor mass signal pattern indicated in red on the right side of each Dha product signal showed the formation of +CN product, Ub-G76C(CN)-6H. The mass signal pattern indicated in blue in the middle mass diagram that came from the 17.51 min peak determined the formation of +TNB product, Ub-G76C(TNB)-6H. The mass signal pattern in the bottom mass diagram that came from the 19.73 min peak designated the formation of hydrolysis product, Ub$_{(1-75)}$.

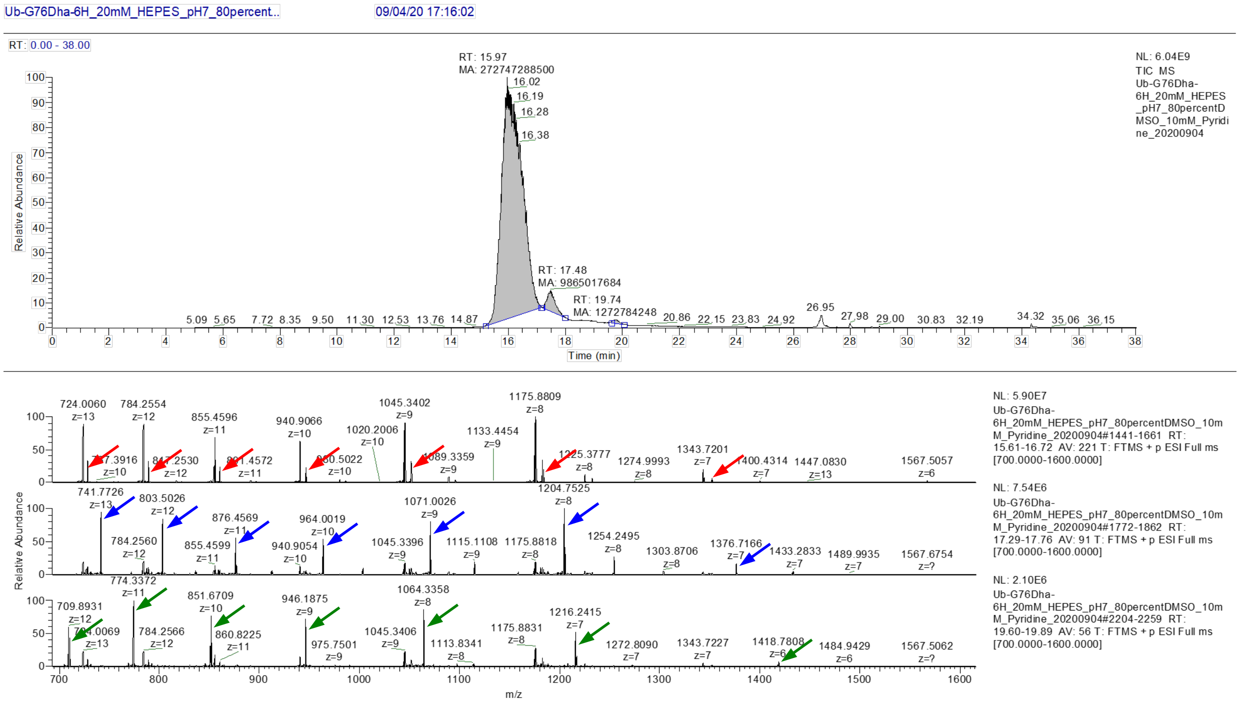

Figure S13. ESI-LC-MS analysis of product mixture of NTCB induced Dha formation reaction using Ub-G76C-6H as the substrate. Reaction condition: 20 mM HEPES pH 7, 80% DMSO, 37 °C, 18 h incubation. The major mass signal pattern in the top mass diagram that came from the 15.97 min peak represented Ub-G76Dha-6H. The minor mass signal pattern indicated in red on the right side of each Dha product signal showed the formation of +CN product, Ub-G76C(CN)-6H. The mass signal pattern indicated in blue in the middle mass diagram that came from the 17.48 min peak determined the formation of +TNB product, Ub-G76C(TNB)-6H. The mass signal pattern indicated in green in the bottom mass diagram that came from the 19.74 min peak designated the formation of hydrolysis product, $Ub_{(1-75)}$.

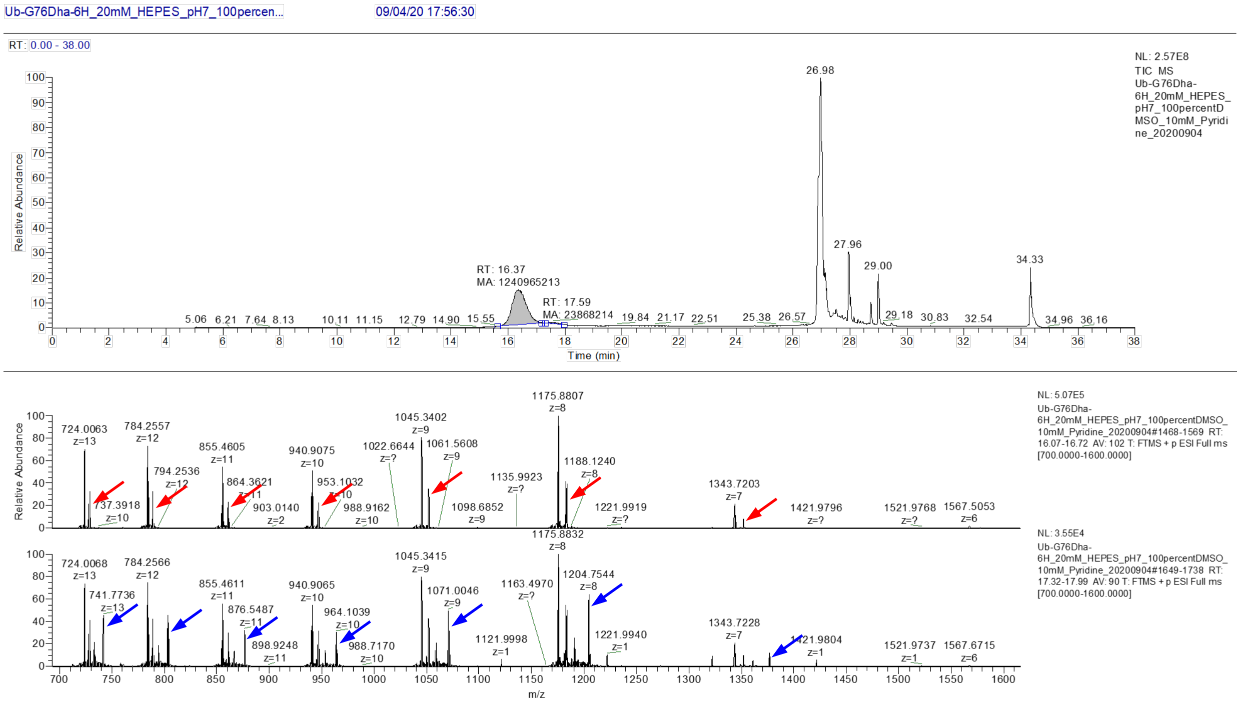

Figure S14. ESI-LC-MS analysis of product mixture of NTCB induced Dha formation reaction using Ub-G76C-6H as the substrate. Reaction condition: 20 mM HEPES pH 7, 100% DMSO, 37 °C, 18 h incubation. The major mass signal pattern in the top mass diagram that came from the 16.37 min peak represented Ub-G76Dha-6H. The minor mass signal pattern indicated in red on the right side of each Dha product signal showed the formation of +CN product, Ub-G76C(CN)-6H. The mass signal pattern indicated in blue in the bottom mass diagram that came from the tailing area of the Dha peak determined the formation of +TNB product, Ub-G76C(TNB)-6H. No hydrolysis product was detected due to the lack of water as solvent.

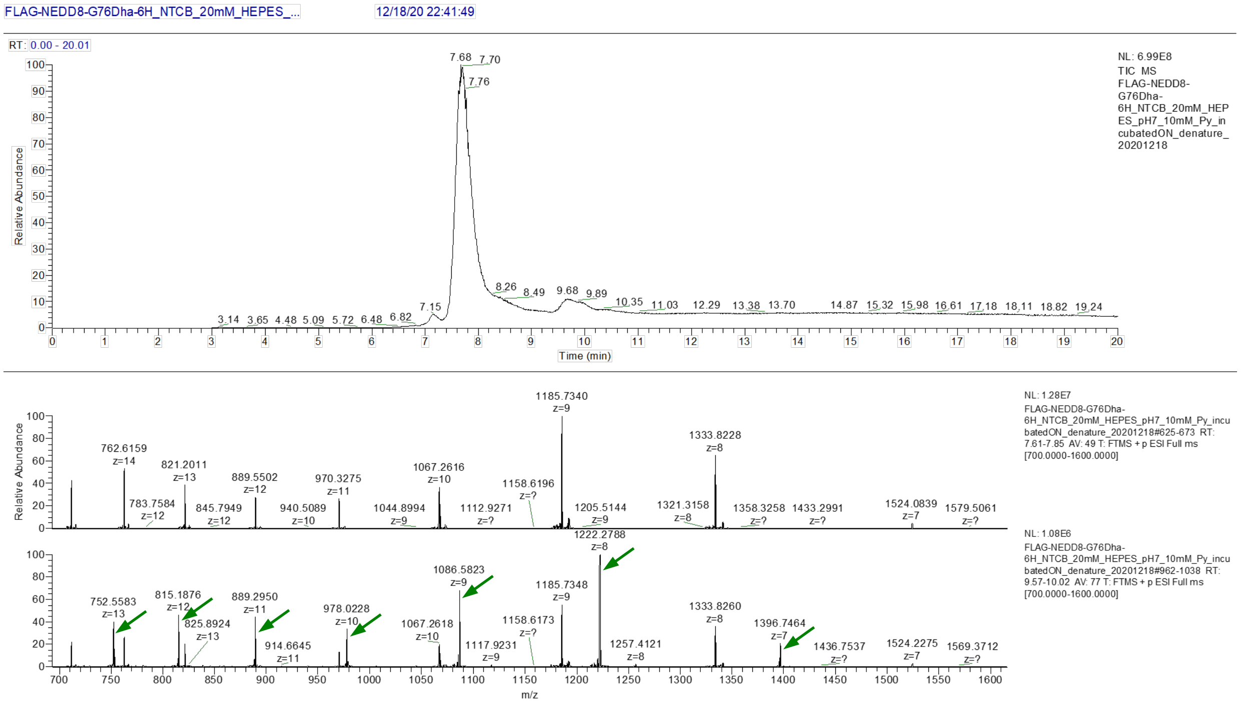

Figure S15. ESI-LC-MS analysis of product mixture of NTCB induced Dha formation reaction using FLAG-NEDD8-G76C-6H as the substrate. Reaction condition: 20 mM HEPES pH 7, 6 M GndCl, 37 °C, 18 h incubation. The mass signal pattern in the top mass diagram that came from the 7.68 min peak represented FLAG-NEDD8-G76Dha-6H. The mass signal pattern indicated in green in the bottom mass diagram that came from the 9.68 min peak showed the formation of hydrolysis product, FLAG-NEDD8$_{(1\text{-}75)}$.

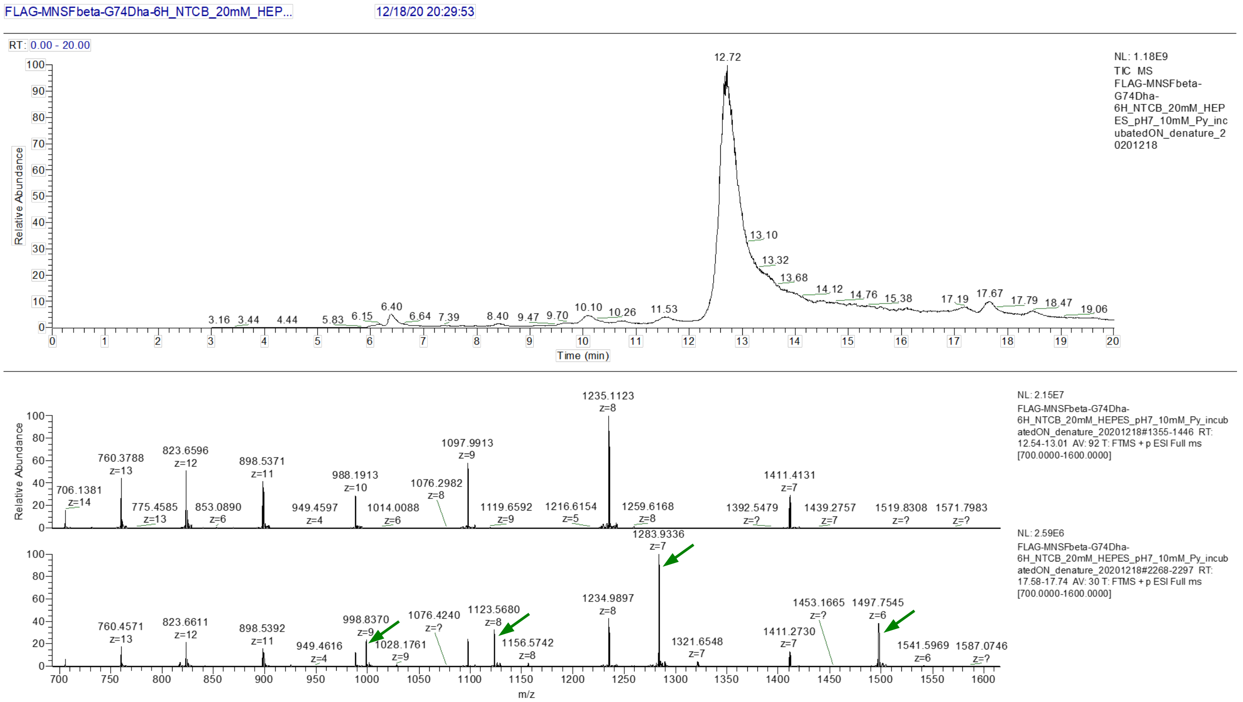

Figure S16. ESI-LC-MS analysis of product mixture of NTCB induced Dha formation reaction using FLAG-MNSFβ-C57S-G74C-6H as the substrate. Reaction condition: 20 mM HEPES pH 7, 6 M GndCl, 37 °C, 18 h incubation. The mass signal pattern in the top mass diagram that came from the 12.72 min peak represented FLAG-MNSFβ-C57S-G74Dha-6H. The mass signal pattern indicated in green in the bottom mass diagram that came from the 17.67 min peak showed the formation of hydrolysis product, FLAG-MNSFβ$_{(1-73)}$-C57S.

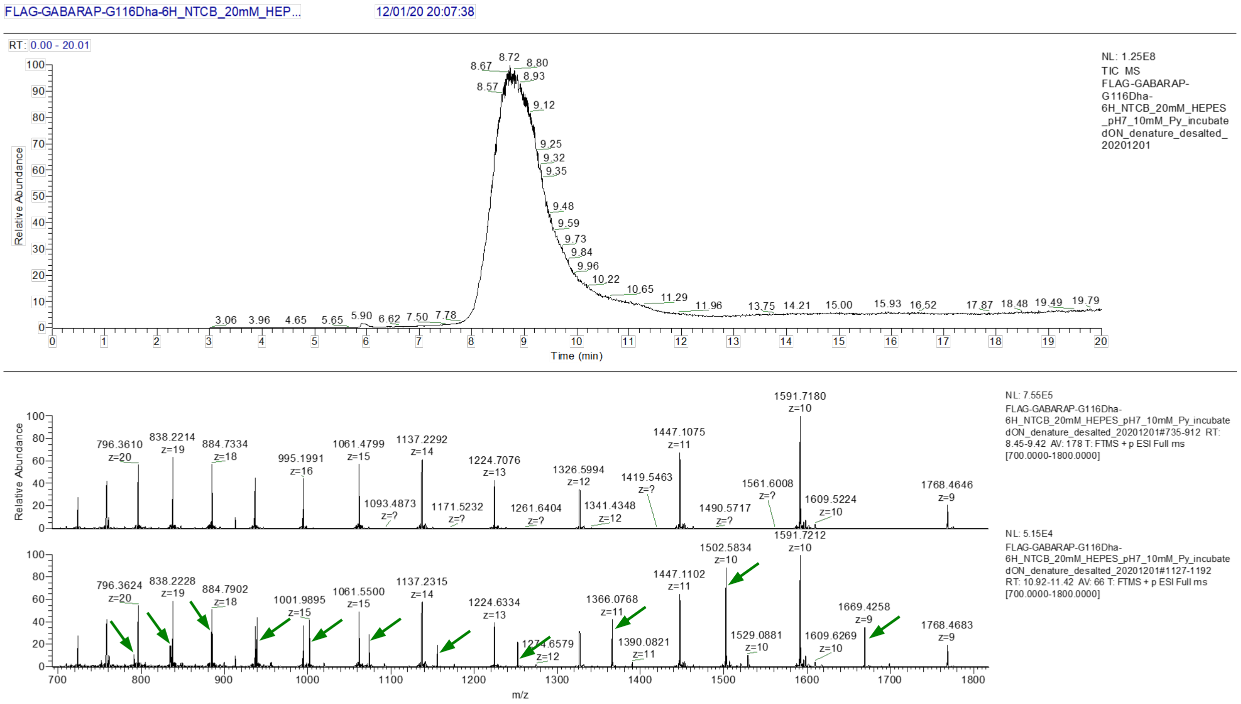

Figure S17. ESI-LC-MS analysis of product mixture of NTCB induced Dha formation reaction using FLAG-GABARAP-G116C-6H as the substrate. Reaction condition: 20 mM HEPES pH 7, 6 M GndCl, 37 °C, 18 h incubation. The mass signal pattern in the top mass diagram that came from the 8.72 min peak represented FLAG-GABARAP-G116Dha-6H. The mass signal pattern indicated in green in the bottom mass diagram that came from the tailing area of Dha product peak showed the formation of hydrolysis product, FLAG-GABARAP$_{(1\text{-}115)}$.

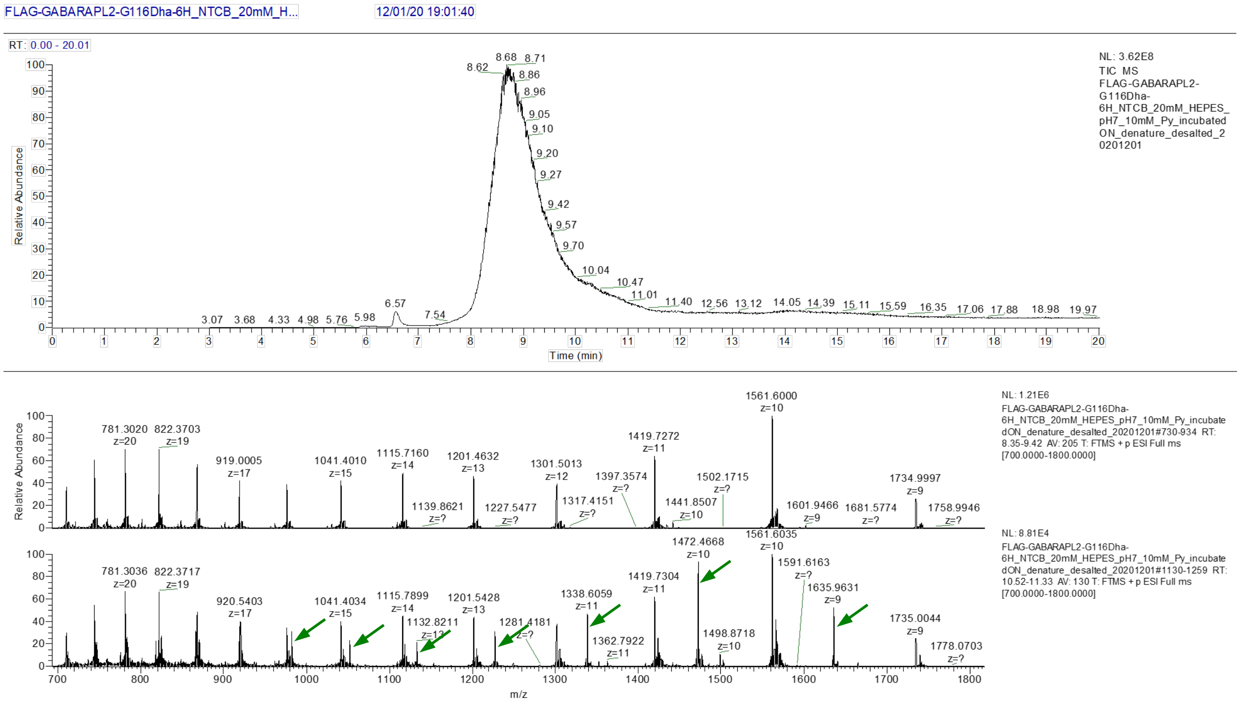

Figure S18. ESI-LC-MS analysis of product mixture of NTCB induced Dha formation reaction using FLAG-GABARAPL2-G116C-6H as the substrate. Reaction condition: 20 mM HEPES pH 7, 6 M GndCl, 37 °C, 18 h incubation. The mass signal pattern in the top mass diagram that came from the 8.68 min peak represented FLAG-GABARAPL2-G116Dha-6H. The mass signal pattern indicated in green in the bottom mass diagram that came from the tailing area of Dha product peak showed the formation of hydrolysis product, FLAG-GABARAPL2$_{(1-115)}$.

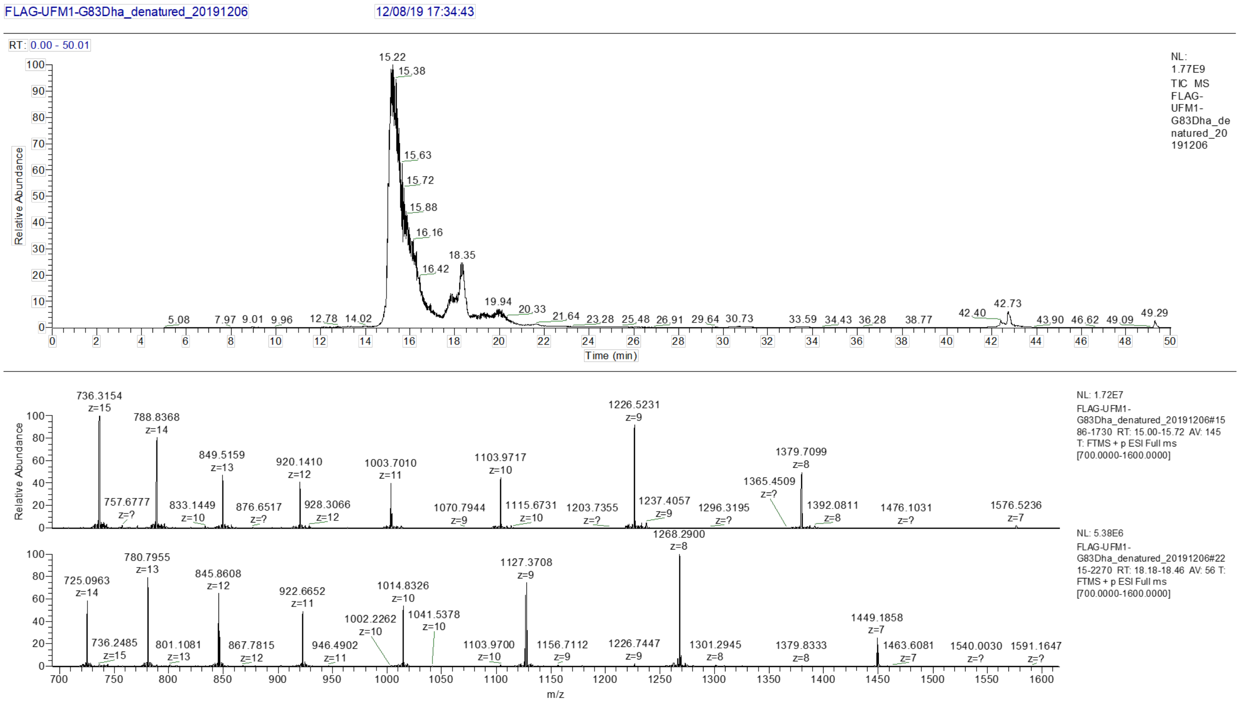

Figure S19. ESI-LC-MS analysis of product mixture of NTCB induced Dha formation reaction using FLAG-UFM1-G83C-6H as the substrate. Reaction condition: 20 mM HEPES pH 7, 6 M GndCl, 37 °C, 18 h incubation. The mass signal pattern in the top mass diagram that came from the 15.22 min peak represented FLAG-UFM1-G83Dha-6H. The mass signal pattern in the bottom mass diagram that came from the 18.35 min peak showed the formation of hydrolysis product, FLAG-UFM1$_{(1\text{-}82)}$.

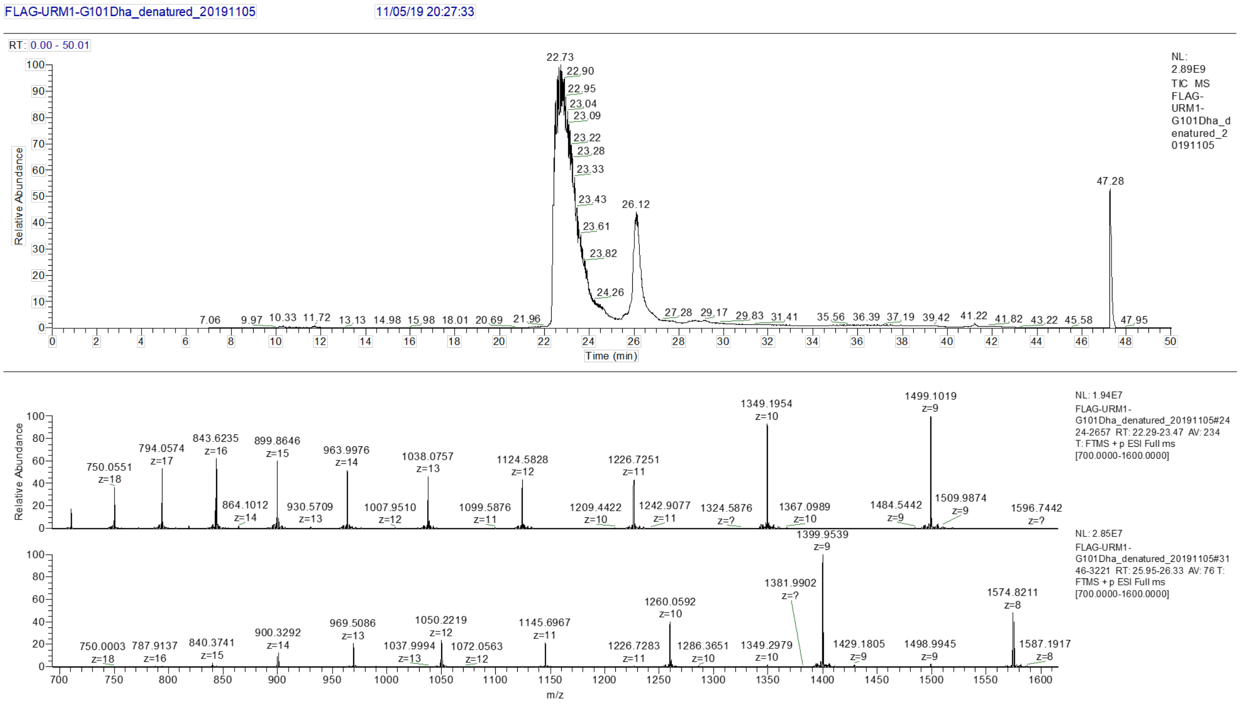

Figure S20. ESI-LC-MS analysis of product mixture of NTCB induced Dha formation reaction using FLAG-URM1-G101C-6H as the substrate. Reaction condition: 20 mM HEPES pH 7, 6 M GndCl, 37 °C, 18 h incubation. The mass signal pattern in the top mass diagram that came from the 22.73 min peak represented FLAG-URM1-G101Dha-6H. The mass signal pattern in the bottom mass diagram that came from the 26.12 min peak showed the formation of hydrolysis product, FLAG-URM1$_{(1\text{-}100)}$.

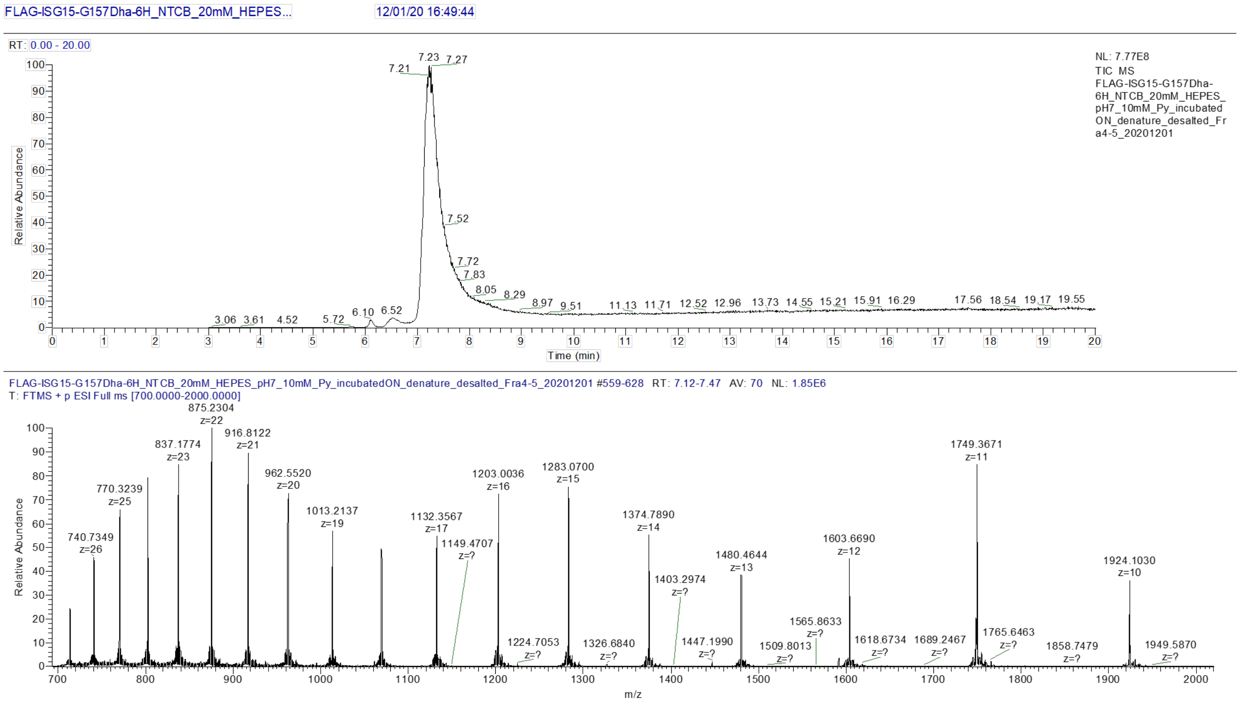

Figure S21. ESI-LC-MS analysis of product mixture of NTCB induced Dha formation reaction using FLAG-ISG15-C89S-G157C-6H as the substrate. Reaction condition: 20 mM HEPES pH 7, 6 M GndCl, 37 °C, 18 h incubation. The mass signal pattern that came from the 7.23 min peak represented FLAG- ISG15-C89S-G157Dha-6H. The formation of hydrolysis product was too minor to be detected.

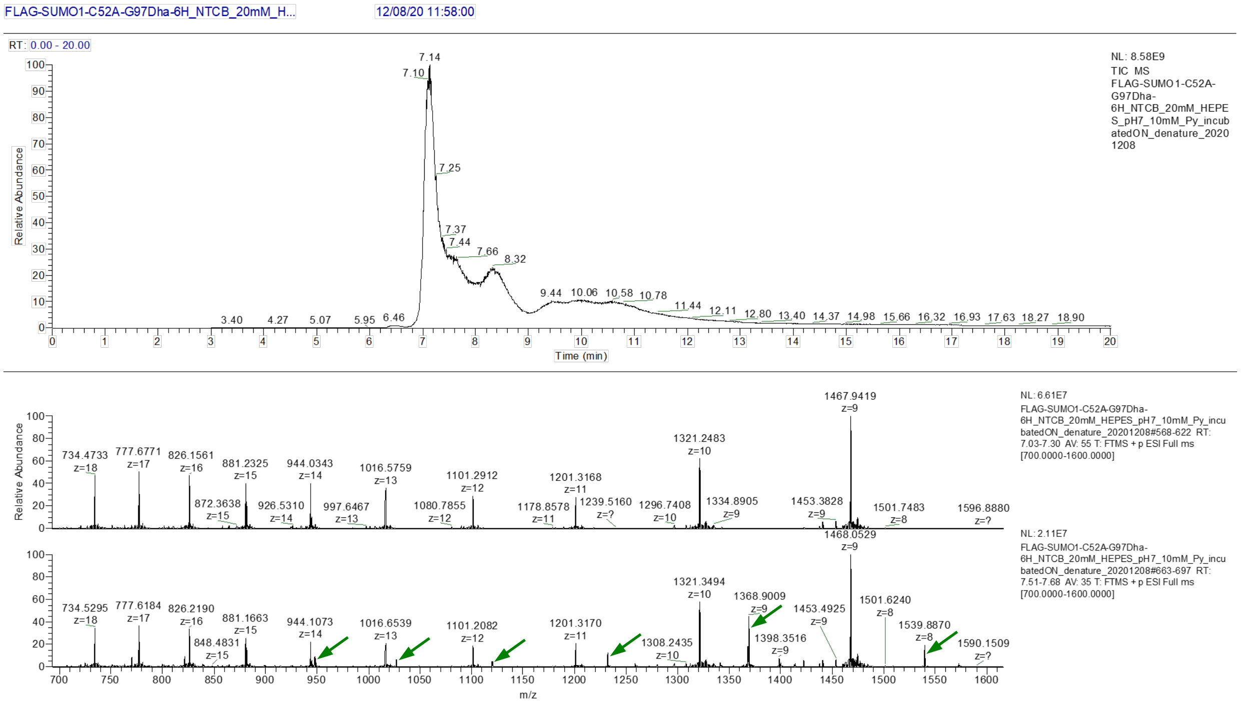

Figure S22. ESI-LC-MS analysis of product mixture of NTCB induced Dha formation reaction using FLAG-SUMO1-C52A-G97C-6H as the substrate. Reaction condition: 20 mM HEPES pH 7, 6 M GndCl, 37 °C, 18 h incubation. The mass signal pattern in the top mass diagram that came from the 7.14 min peak represented FLAG-SUMO1-C52A-G97Dha-6H. The mass signal pattern indicated in green in the bottom mass diagram that came from the tailing area (~7.66 min) of Dha product peak showed the formation of hydrolysis product, FLAG-SUMO1$_{(1\text{-}96)}$-C52A.

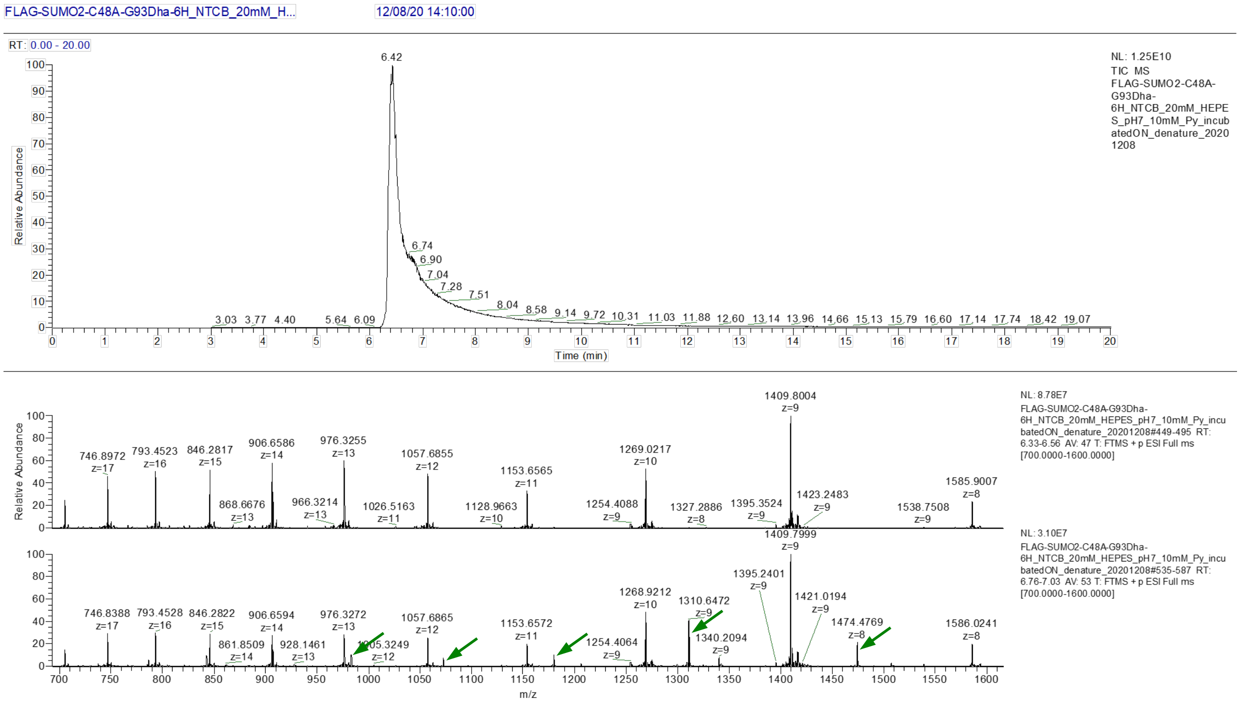

Figure S23. ESI-LC-MS analysis of product mixture of NTCB induced Dha formation reaction using FLAG-SUMO2-C48A-G93C-6H as the substrate. Reaction condition: 20 mM HEPES pH 7, 6 M GndCl, 37 °C, 18 h incubation. The mass signal pattern in the top mass diagram that came from the 6.42 min peak represented FLAG- SUMO2-C48A-G93Dha-6H. The mass signal pattern indicated in green in the bottom mass diagram that came from the tailing area of Dha product peak showed the formation of hydrolysis product, FLAG-SUMO2$_{(1-92)}$-C48A.

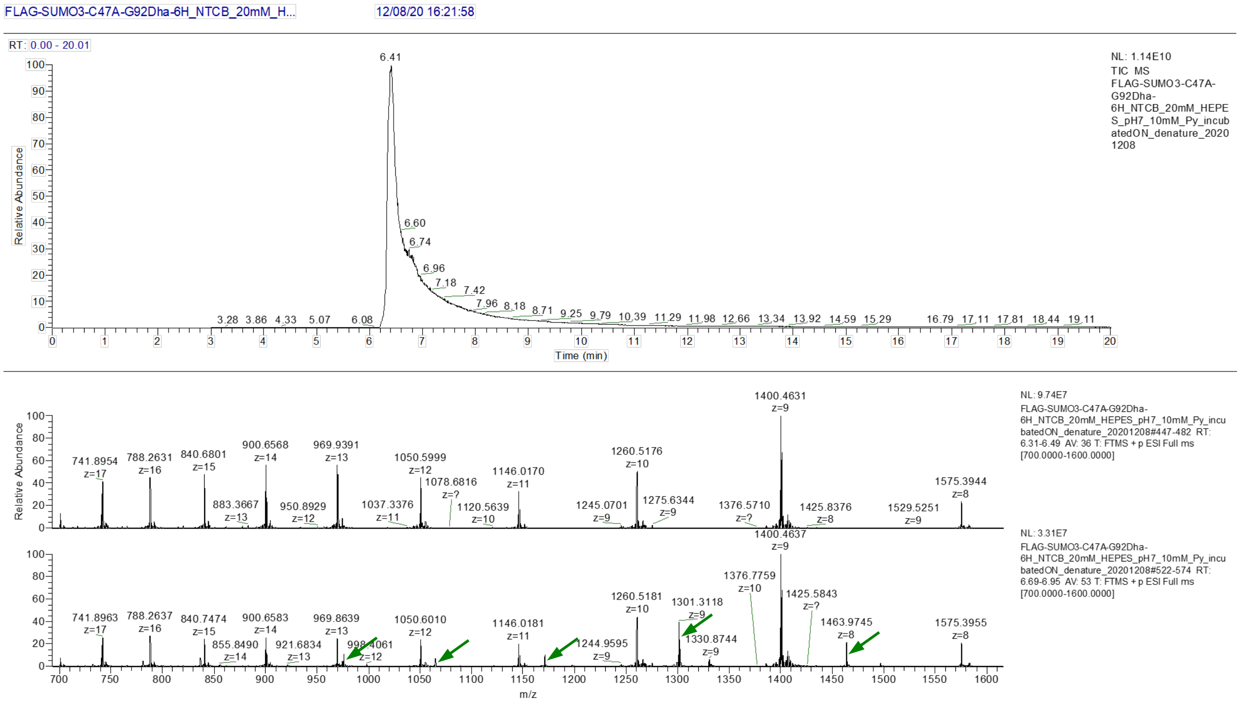

Figure S24. ESI-LC-MS analysis of product mixture of NTCB induced Dha formation reaction using FLAG-SUMO3-C47A-G92C-6H as the substrate. Reaction condition: 20 mM HEPES pH 7, 6 M GndCl, 37 °C, 18 h incubation. The mass signal pattern in the top mass diagram that came from the 6.41 min peak represented FLAG- SUMO3-C47A-G92Dha-6H. The mass signal pattern indicated in green in the bottom mass diagram that came from the tailing area of Dha product peak showed the formation of hydrolysis product, FLAG-SUMO3$_{(1-91)}$-C47A.

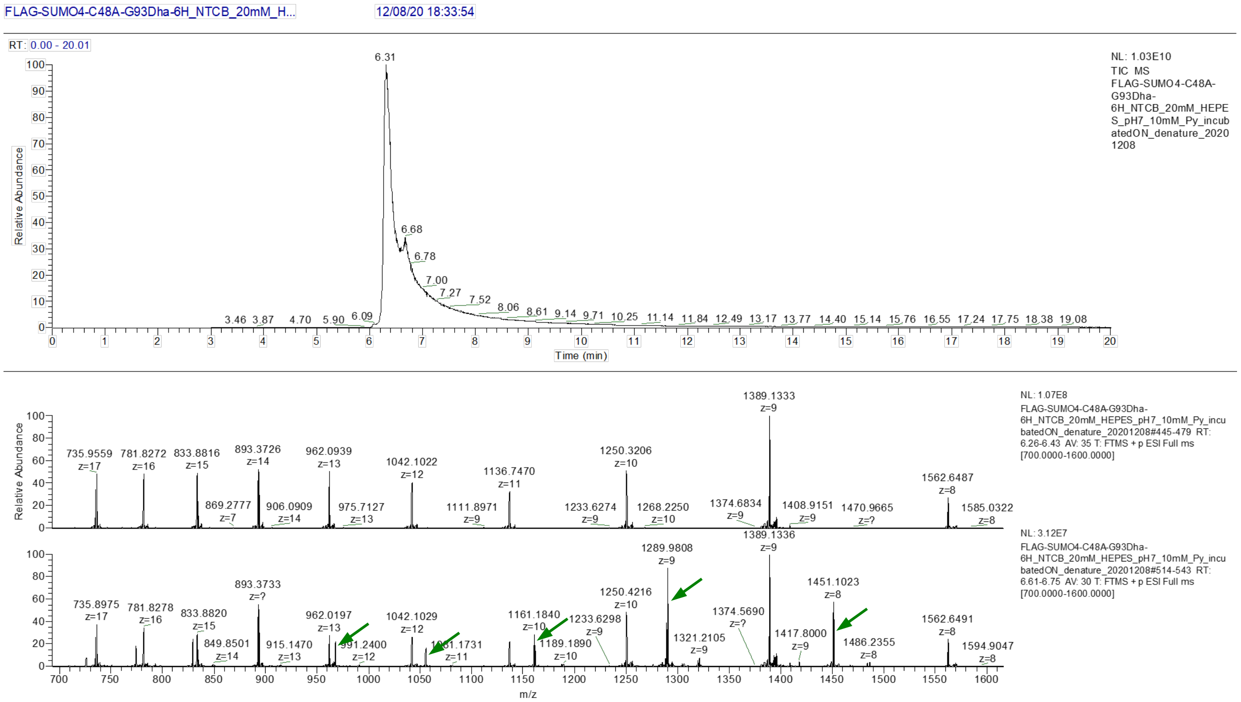

Figure S25. ESI-LC-MS analysis of product mixture of NTCB induced Dha formation reaction using FLAG-SUMO4-C48A-G93C-6H as the substrate. Reaction condition: 20 mM HEPES pH 7, 6 M GndCl, 37 °C, 18 h incubation. The mass signal pattern in the top mass diagram that came from the 6.31 min peak represented FLAG- SUMO4-C48A-G93Dha-6H. The mass signal pattern indicated in green in the bottom mass diagram that came from the tailing area (~6.68 min) of Dha product peak showed the formation of hydrolysis product, FLAG-SUMO4$_{(1-92)}$-C48A.

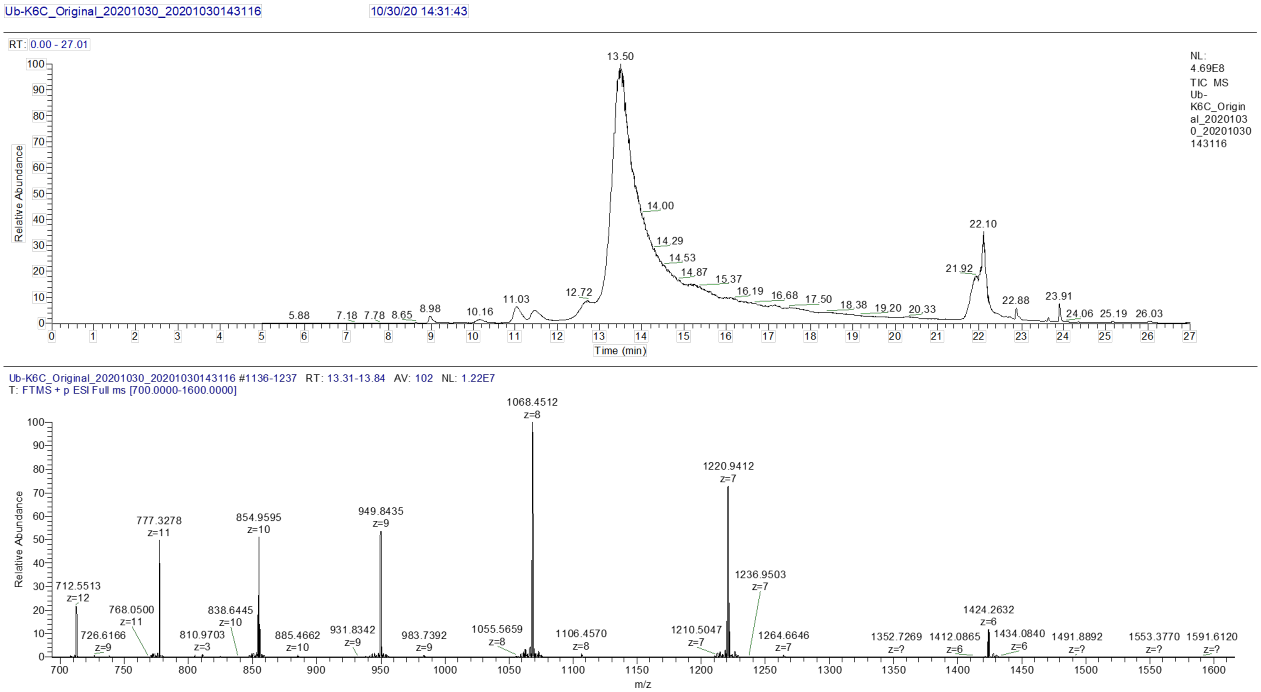

Figure S26. ESI-LC-MS analysis and deconvoluted spectra of purified Ub-K6C.

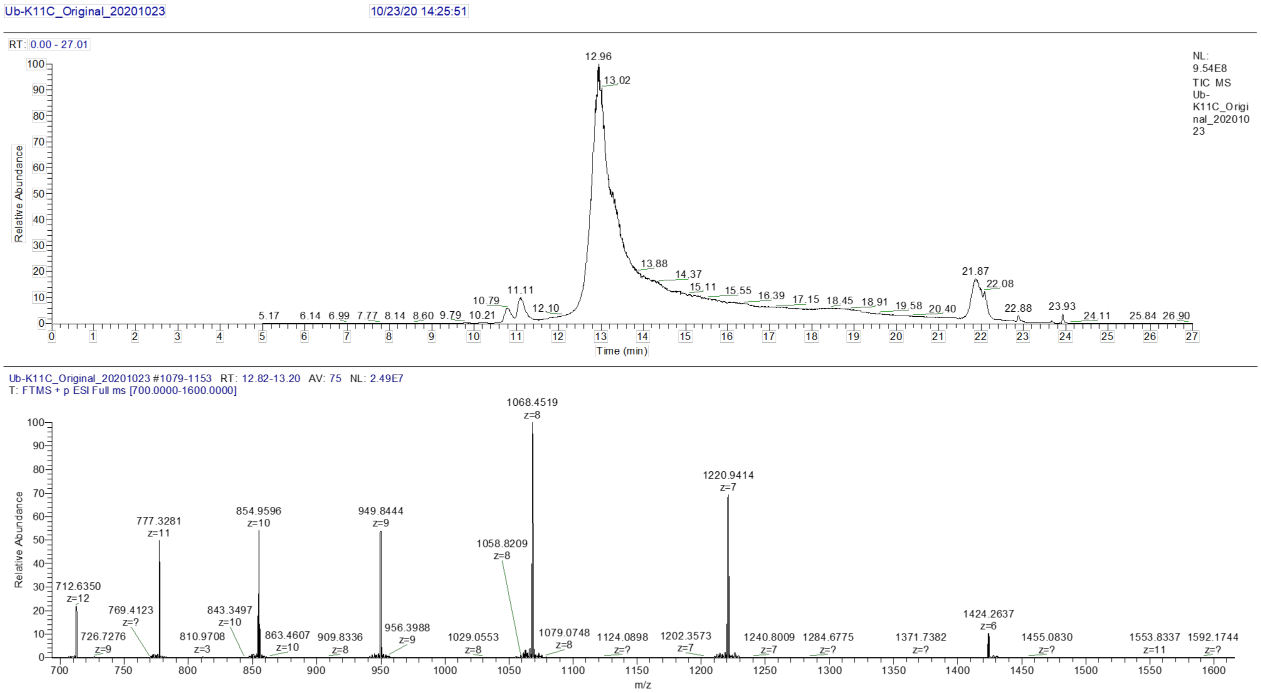

Figure S27. ESI-LC-MS analysis and deconvoluted spectra of purified Ub-K11C.

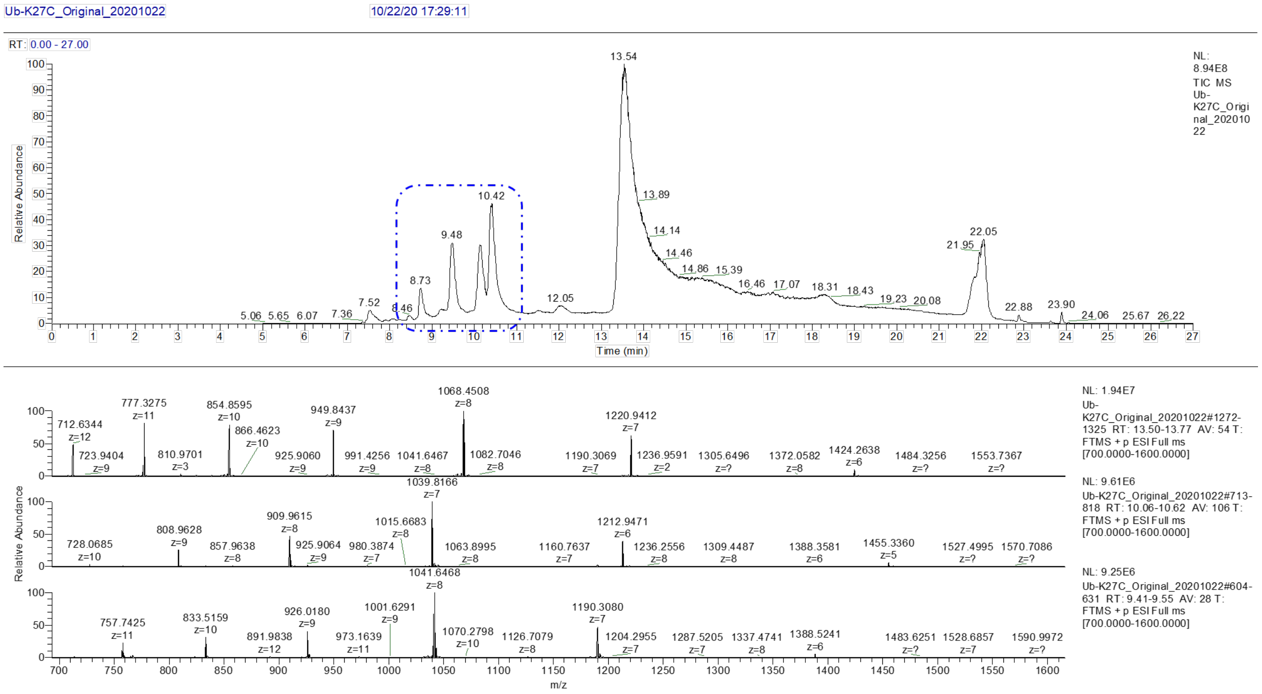

Figure S28. ESI-LC-MS analysis and deconvoluted spectra of purified Ub-K27C. Blue dash line circled peaks referred to small molecular weight impurities that were inactive to NTCB as indicated by the bottom two mass diagrams.

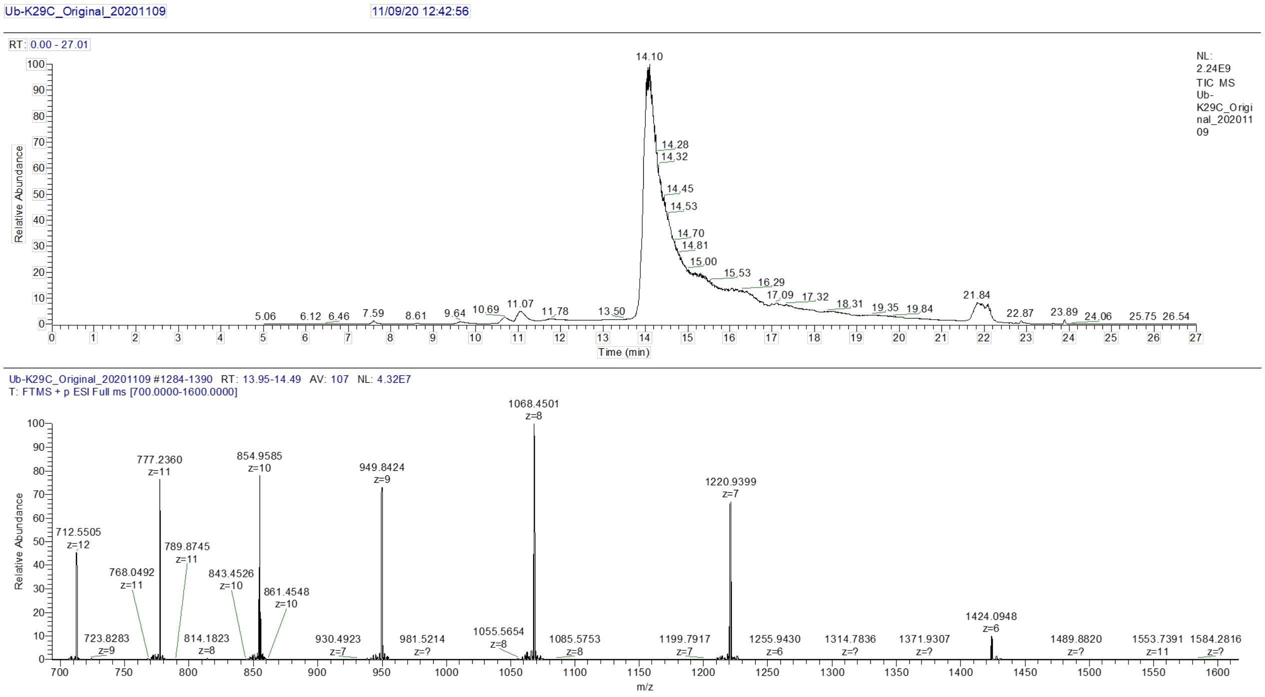

Figure S29. ESI-LC-MS analysis and deconvoluted spectra of purified Ub-K29C.

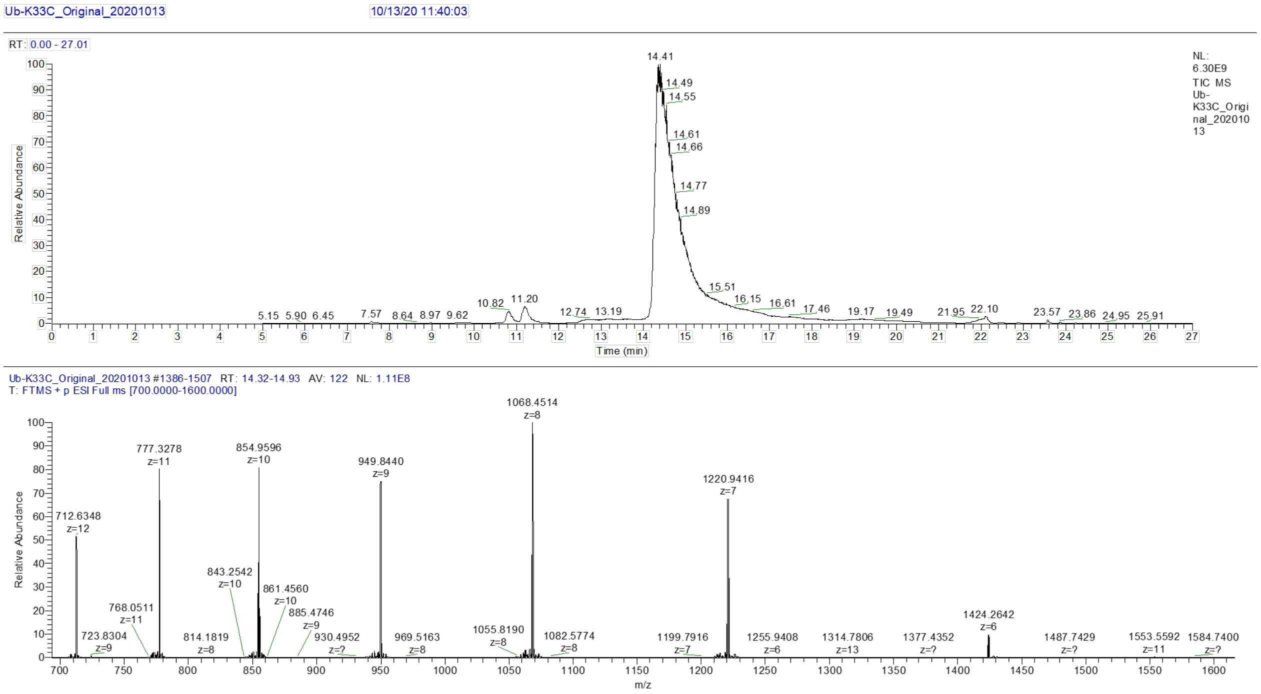

Figure S30. ESI-LC-MS analysis and deconvoluted spectra of purified Ub-K33C.

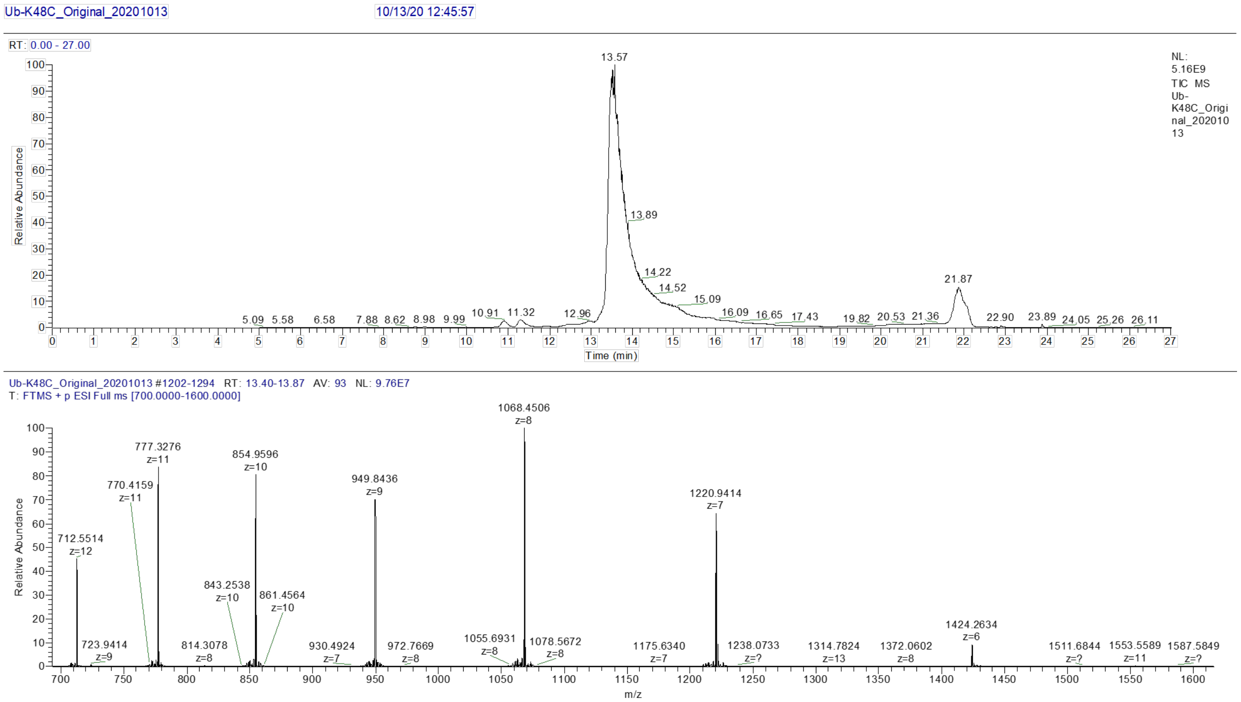

Figure S31. ESI-LC-MS analysis and deconvoluted spectra of purified Ub-K48C.

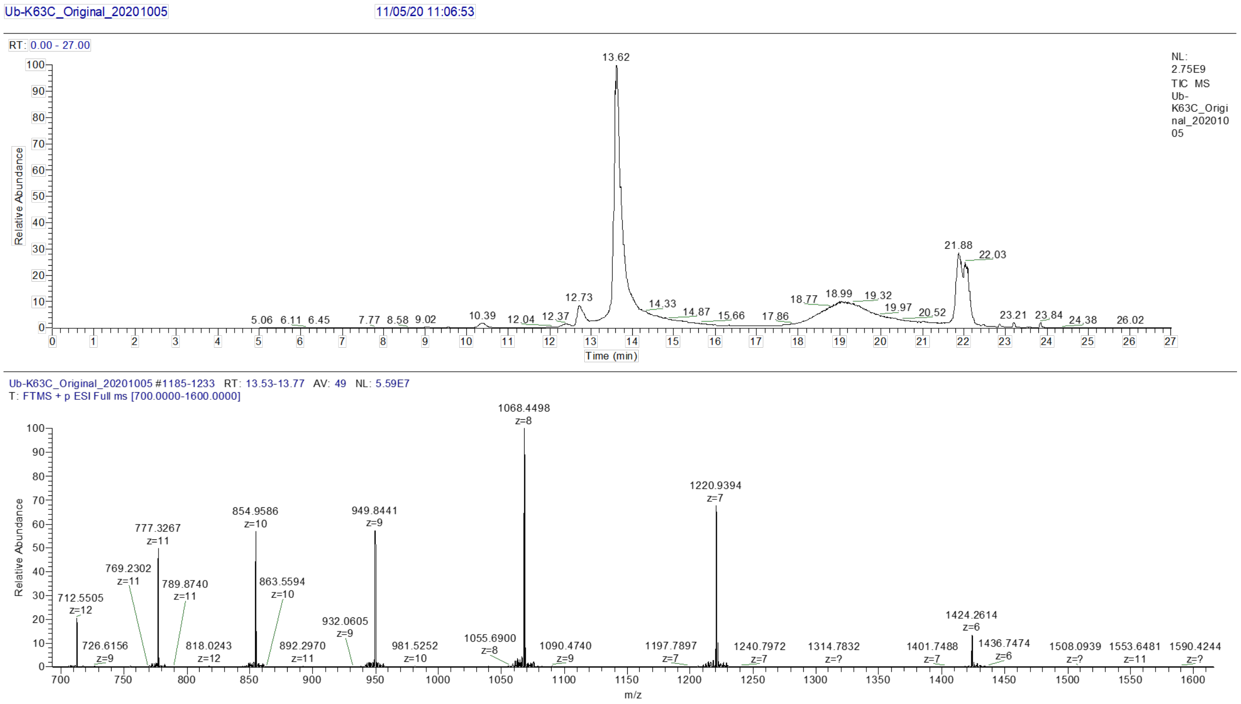

Figure S32. ESI-LC-MS analysis and deconvoluted spectra of purified Ub-K63C.

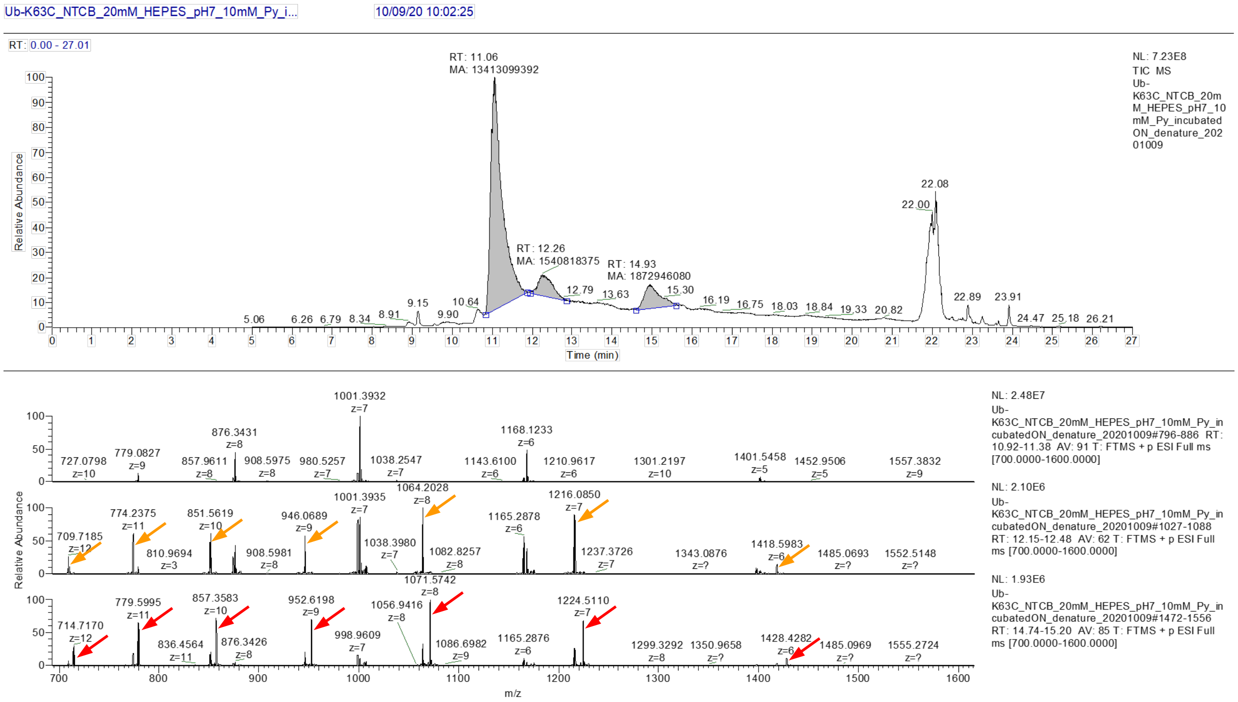

Figure S33. ESI-LC-MS analysis of product mixture of NTCB induced Dha formation reaction using Ub-K63C as the substrate. Reaction condition: 20 mM HEPES pH 7, 6 M GndCl, 37 °C, 18 h incubation. The mass signal pattern in the top mass diagram that came from the 11.06 min peak determined the formation of hydrolysis product, $Ub_{(1-62)}$. The mass signal pattern indicated in orange in the middle mass diagram that came from the 12.26 min peak represented Ub-K63Dha. The mass signal pattern indicated in red in the bottom mass diagram that came from the 14.93 min peak showed the formation of +CN product, Ub-K63C(CN).

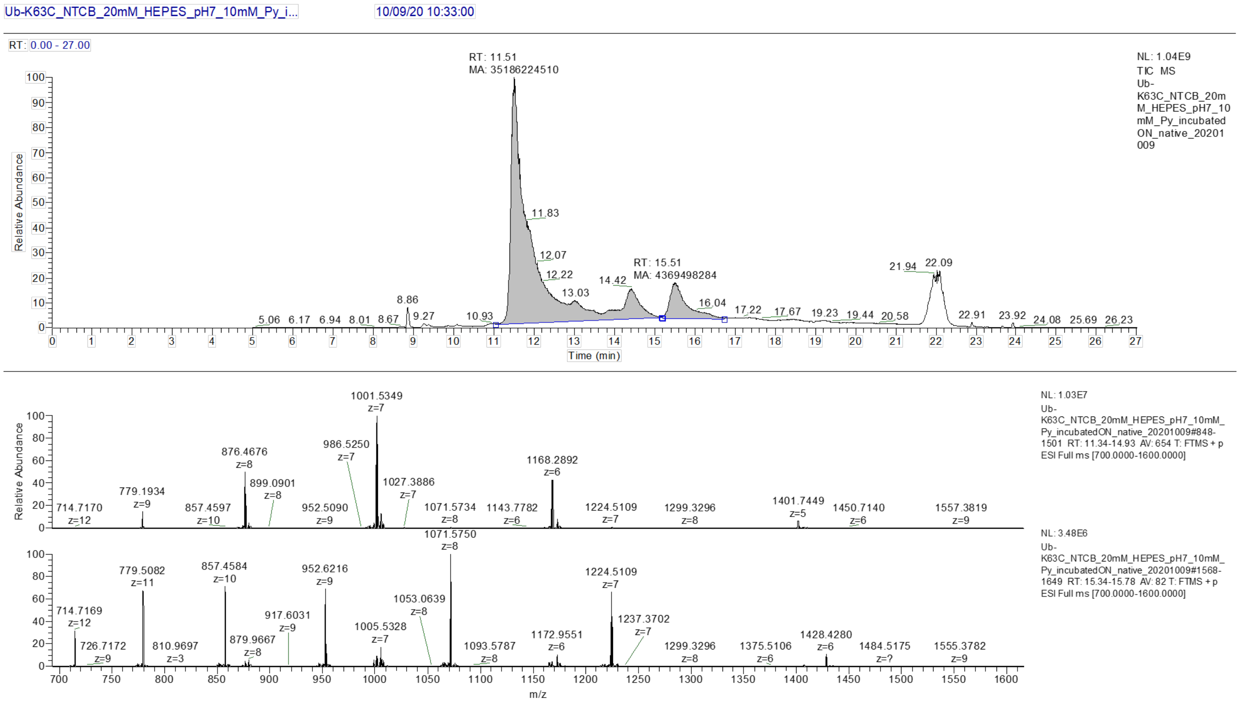

Figure S34. ESI-LC-MS analysis of product mixture of NTCB induced Dha formation reaction using Ub-K63C as the substrate. Reaction condition: 20 mM HEPES pH 7, 37 °C, 18 h incubation. The mass signal pattern in the top mass diagram that came from the 11.51 min peak determined the formation of hydrolysis product, Ub$_{(1-62)}$. The mass signal pattern in the bottom mass diagram that came from the 15.51 min peak showed the formation of +CN product, Ub-K63C(CN).

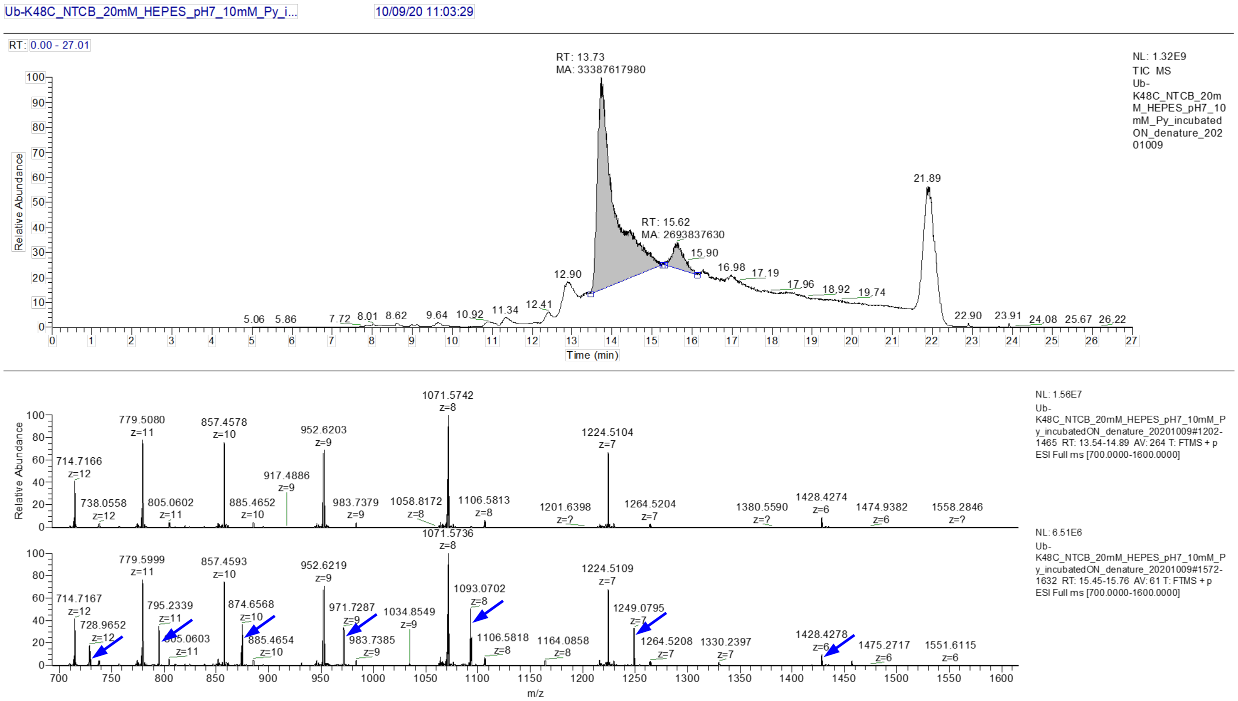

Figure S35. ESI-LC-MS analysis of product mixture of NTCB induced Dha formation reaction using Ub-K48C as the substrate. Reaction condition: 20 mM HEPES pH 7, 6 M GndCl, 37 °C, 18 h incubation. The mass signal pattern in the top mass diagram that came from the 13.73 min peak represented +CN product, Ub-K48C(CN). The mass signal pattern in the bottom mass diagram indicated in blue that came from the 15.62 min peak showed the formation of +TNB product, Ub-K48C(TNB).

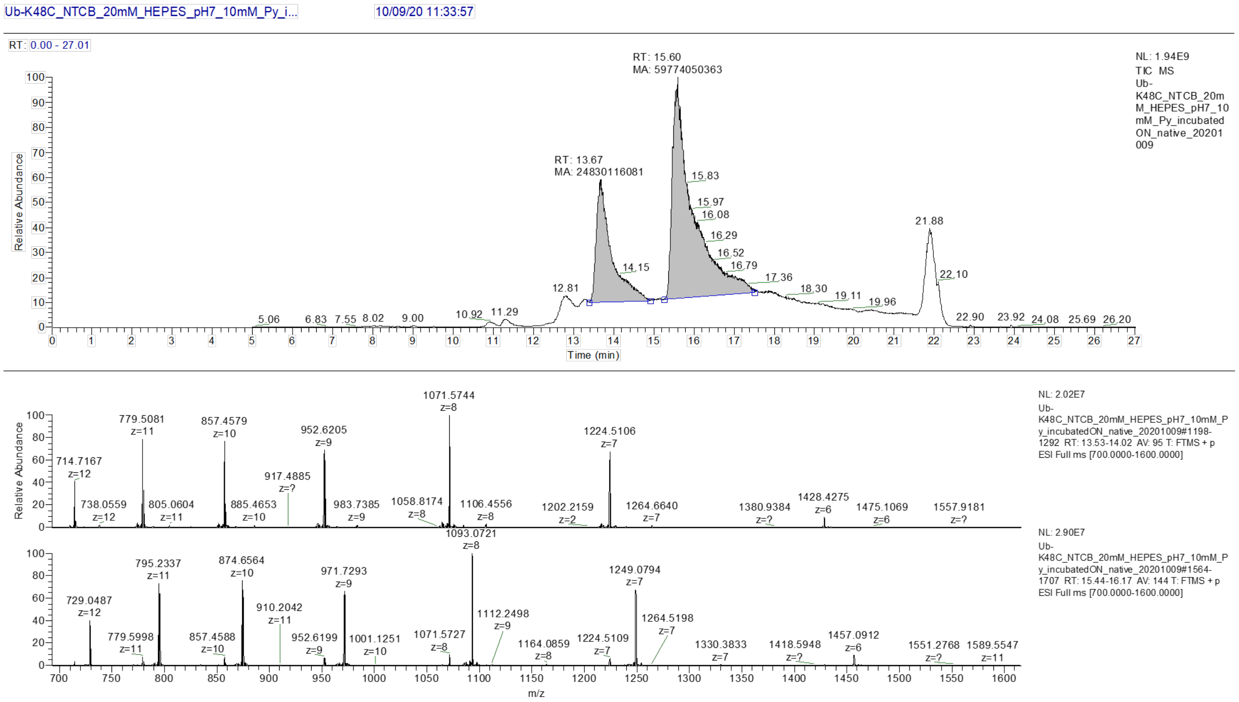

Figure S36. ESI-LC-MS analysis of product mixture of NTCB induced Dha formation reaction using Ub-K48C as the substrate. Reaction condition: 20 mM HEPES pH 7, 37 °C, 18 h incubation. The mass signal pattern in the top mass diagram that came from the 13.67 min peak represented +CN product, Ub-K48C(CN). The mass signal pattern in the bottom mass diagram that came from the 15.60 min peak showed the formation of +TNB product, Ub-K48C(TNB).

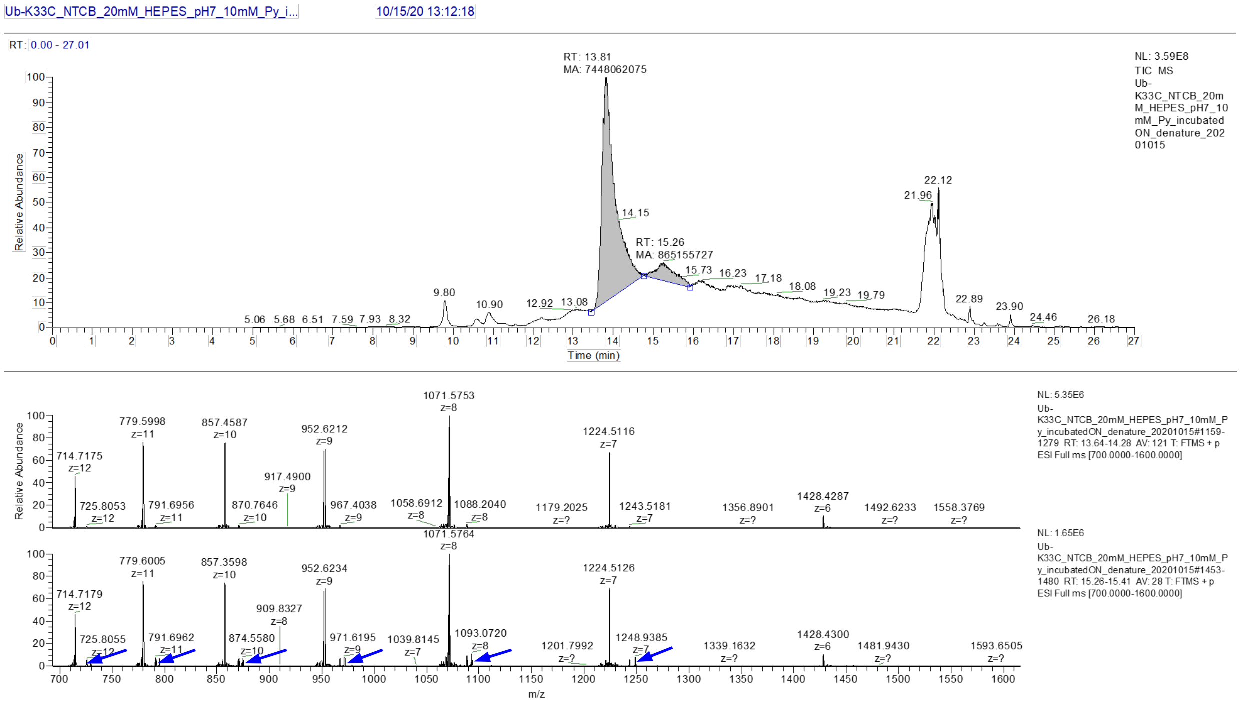

Figure S37. ESI-LC-MS analysis of product mixture of NTCB induced Dha formation reaction using Ub-K33C as the substrate. Reaction condition: 20 mM HEPES pH 7, 6 M GndCl, 37 °C, 18 h incubation. The mass signal pattern in the top mass diagram that came from the 13.81 min peak represented +CN product, Ub-K33C(CN). The mass signal pattern in the bottom mass diagram indicated in blue that came from the 15.26 min peak showed the formation of +TNB product, Ub-K33C(TNB).

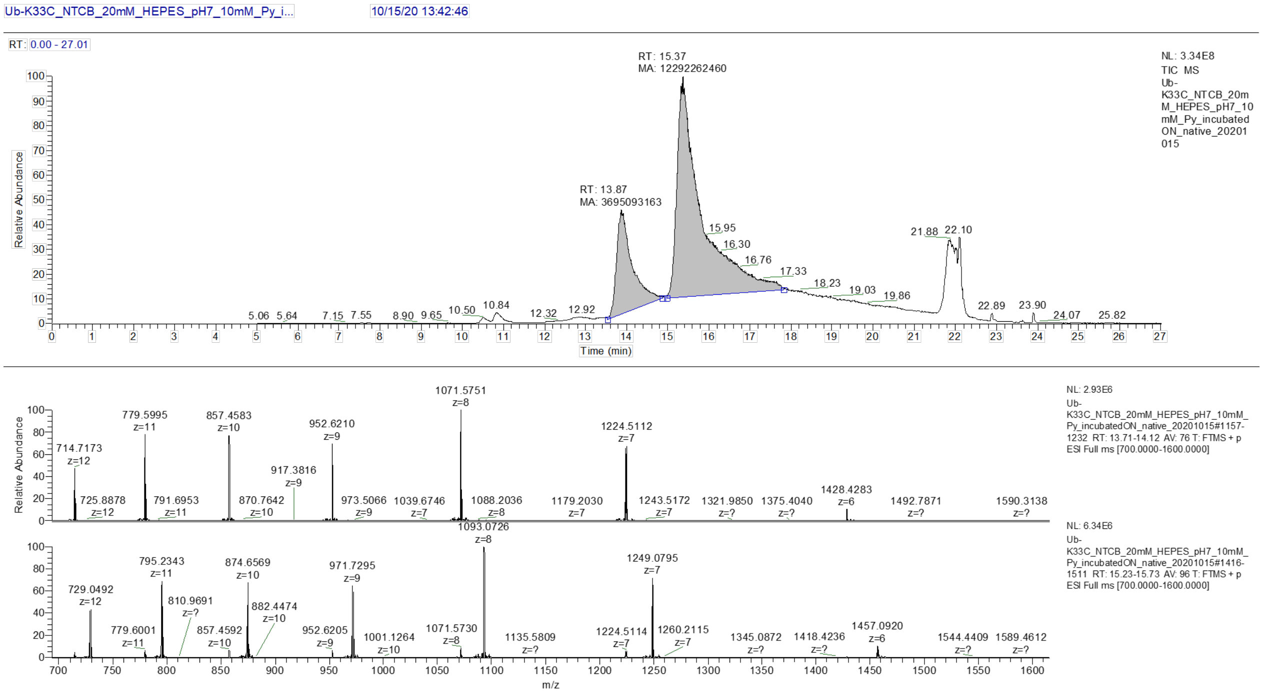

Figure S38. ESI-LC-MS analysis of product mixture of NTCB induced Dha formation reaction using Ub-K33C as the substrate. Reaction condition: 20 mM HEPES pH 7, 37 °C, 18 h incubation. The mass signal pattern in the top mass diagram that came from the 13.87 min peak represented +CN product, Ub-K33C(CN). The mass signal pattern in the bottom mass diagram that came from the 15.37 min peak showed the formation of +TNB product, Ub-K33C(TNB).

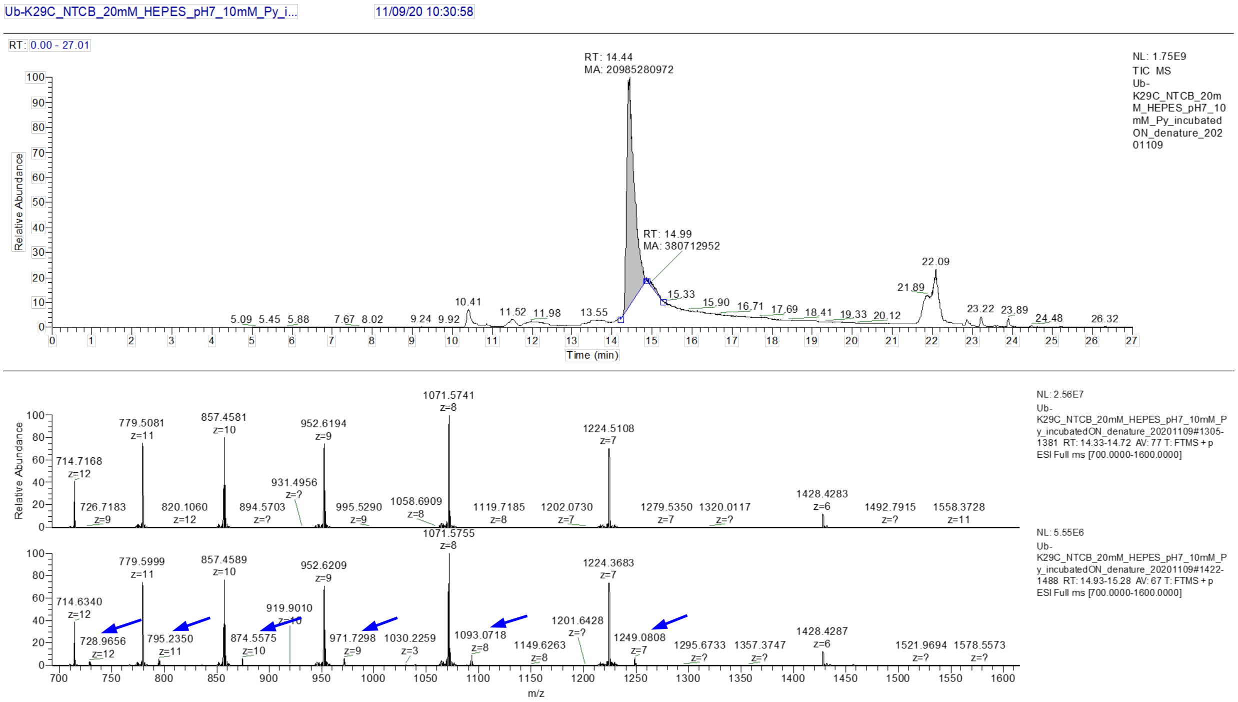

Figure S39. ESI-LC-MS analysis of product mixture of NTCB induced Dha formation reaction using Ub-K29C as the substrate. Reaction condition: 20 mM HEPES pH 7, 6 M GndCl, 37 °C, 18 h incubation. The mass signal pattern in the top mass diagram that came from the 14.44 min peak represented +CN product, Ub-K29C(CN). The mass signal pattern in the bottom mass diagram indicated in blue that came from the 14.99 min peak showed the formation of +TNB product, Ub-K29C(TNB).

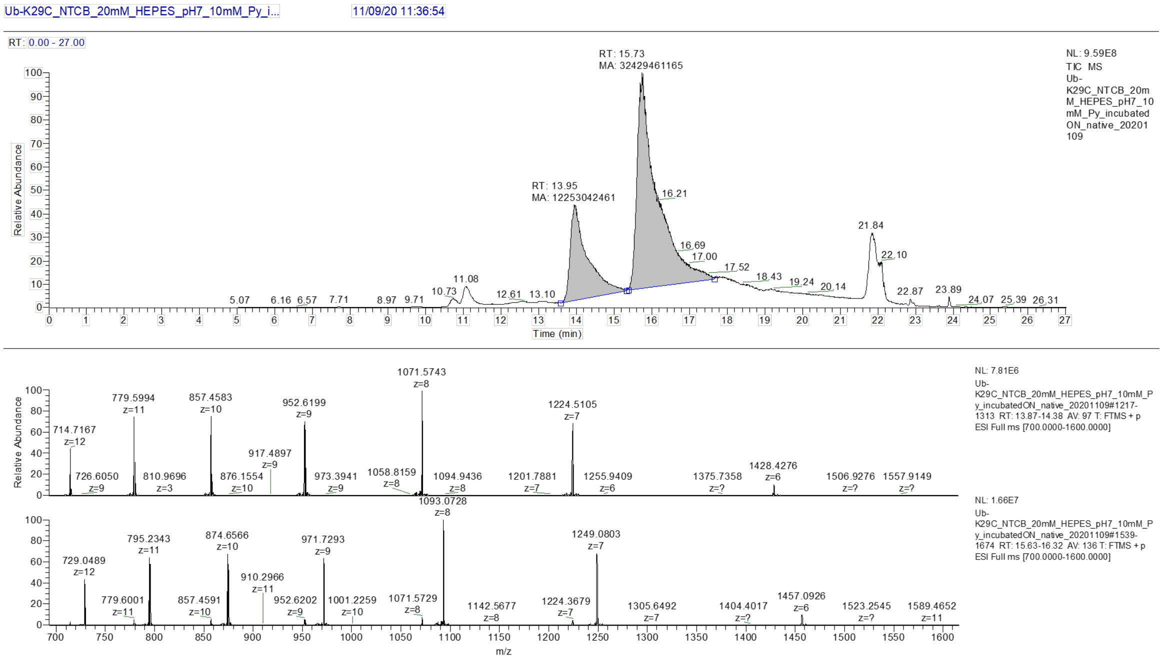

Figure S40. ESI-LC-MS analysis of product mixture of NTCB induced Dha formation reaction using Ub-K29C as the substrate. Reaction condition: 20 mM HEPES pH 7, 37 °C, 18 h incubation. The mass signal pattern in the top mass diagram that came from the 13.95 min peak represented +CN product, Ub-K29C(CN). The mass signal pattern in the bottom mass diagram that came from the 15.73 min peak showed the formation of +TNB product, Ub-K29C(TNB).

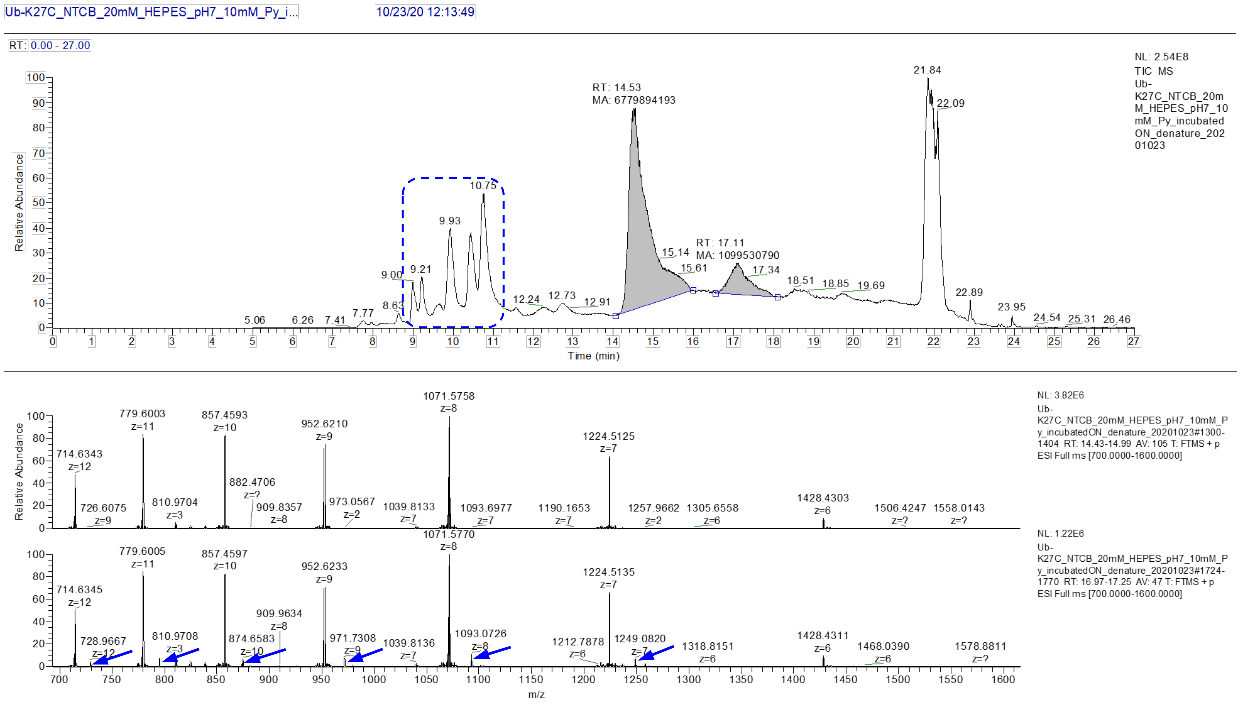

Figure S41. ESI-LC-MS analysis of product mixture of NTCB induced Dha formation reaction using Ub-K27C as the substrate. Reaction condition: 20 mM HEPES pH 7, 6 M GndCl, 37 °C, 18 h incubation. The mass signal pattern in the top mass diagram that came from the 14.53 min peak represented +CN product, Ub-K27C(CN). The mass signal pattern in the bottom mass diagram indicated in blue that came from the 17.11 min peak showed the formation of +TNB product, Ub-K27C(TNB). The dash line circled peaks were unreactive impurities from original Ub-K27C sample.

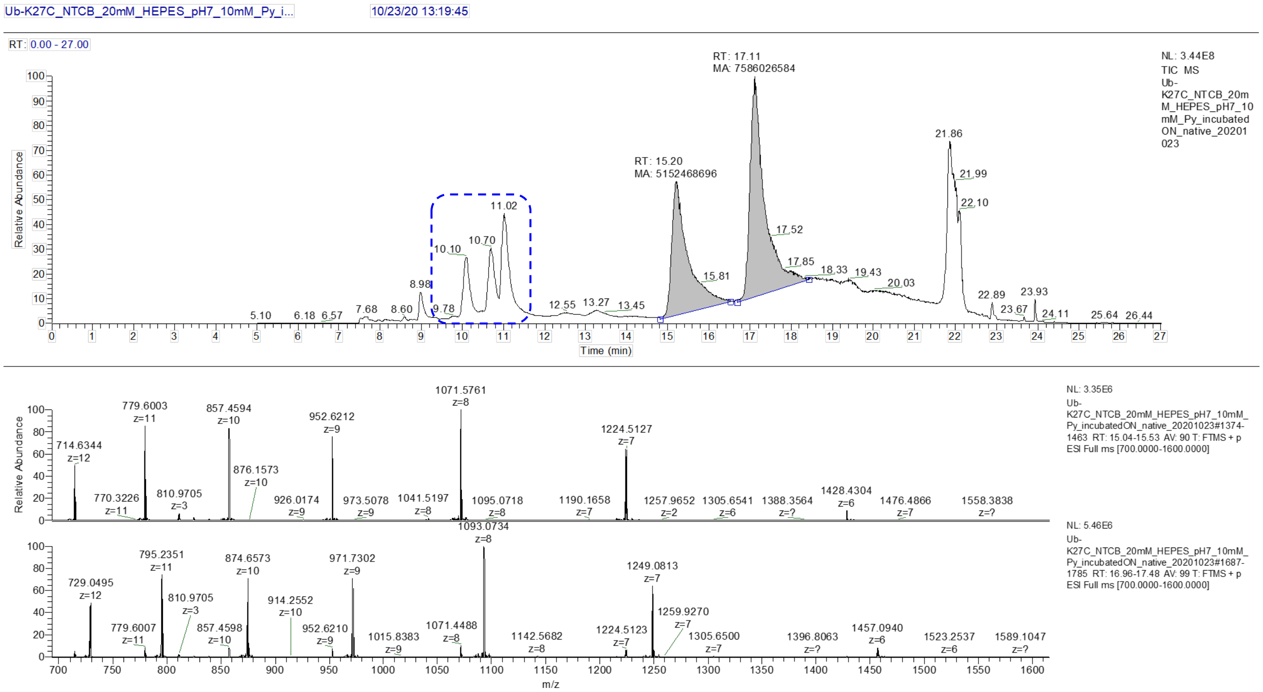

Figure S42. ESI-LC-MS analysis of product mixture of NTCB induced Dha formation reaction using Ub-K27C as the substrate. Reaction condition: 20 mM HEPES pH 7, 37 °C, 18 h incubation. The mass signal pattern in the top mass diagram that came from the 15.20 min peak represented +CN product, Ub-K27C(CN). The mass signal pattern in the bottom mass diagram that came from the 17.11 min peak showed the formation of +TNB product, Ub-K27C(TNB). The dash line circled peaks were unreactive impurities from original Ub-K27C sample.

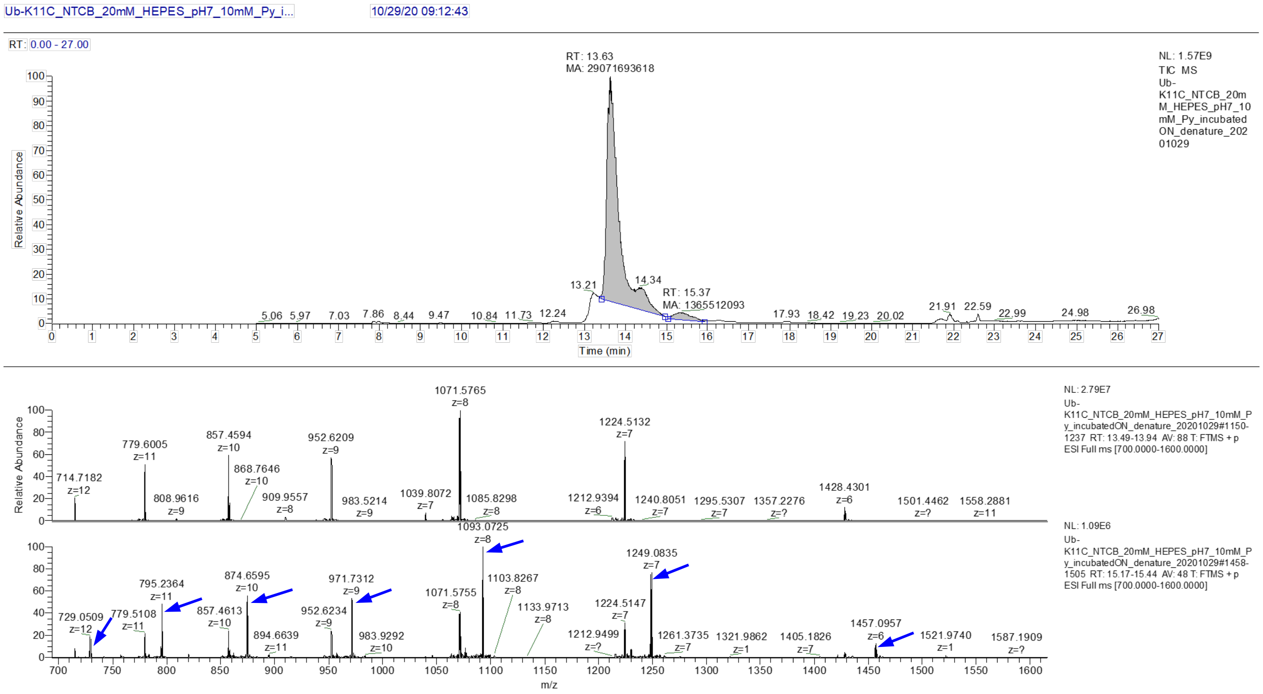

Figure S43. ESI-LC-MS analysis of product mixture of NTCB induced Dha formation reaction using Ub-K11C as the substrate. Reaction condition: 20 mM HEPES pH 7, 6 M GndCl, 37 °C, 18 h incubation. The mass signal pattern in the top mass diagram that came from the 13.63 min peak represented +CN product, Ub-K11C(CN). The mass signal pattern in the bottom mass diagram indicated in blue that came from the 15.37 min peak showed the formation of +TNB product, Ub-K11C(TNB).

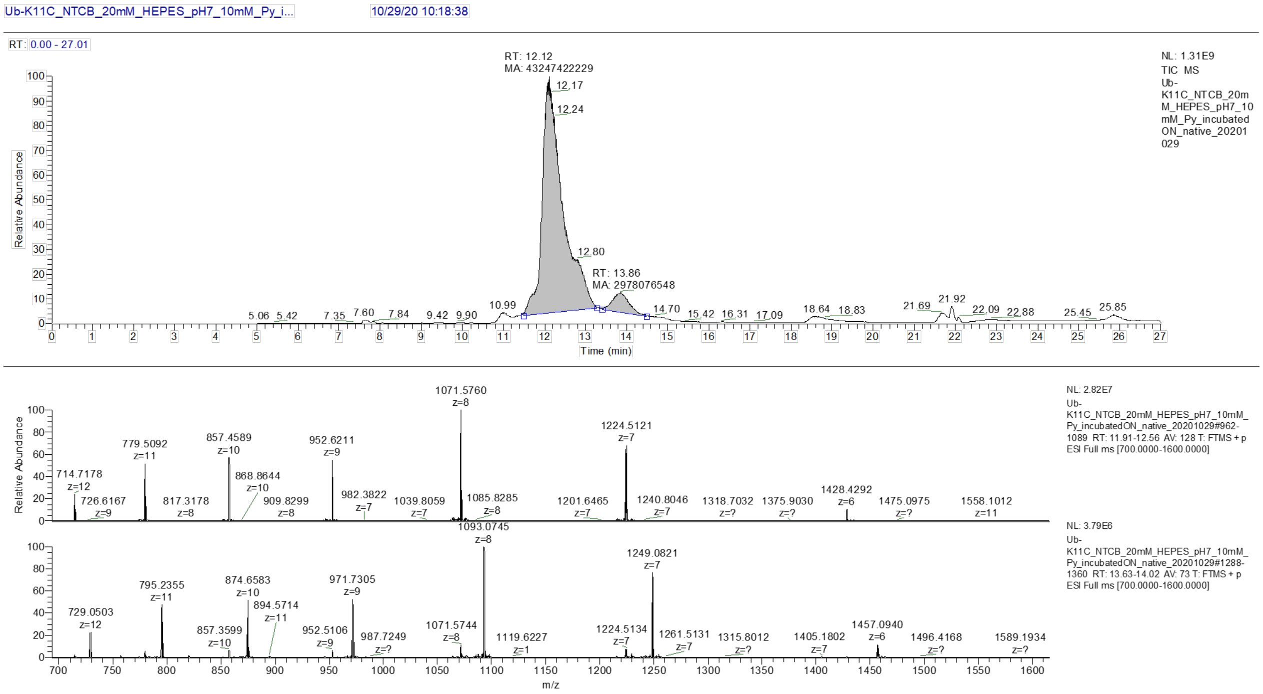

Figure S44. ESI-LC-MS analysis of product mixture of NTCB induced Dha formation reaction using Ub-K11C as the substrate. Reaction condition: 20 mM HEPES pH 7, 37 °C, 18 h incubation. The mass signal pattern in the top mass diagram that came from the 12.12 min peak represented +CN product, Ub-K11C(CN). The mass signal pattern in the bottom mass diagram that came from the 13.86 min peak showed the formation of +TNB product, Ub-K11C(TNB).

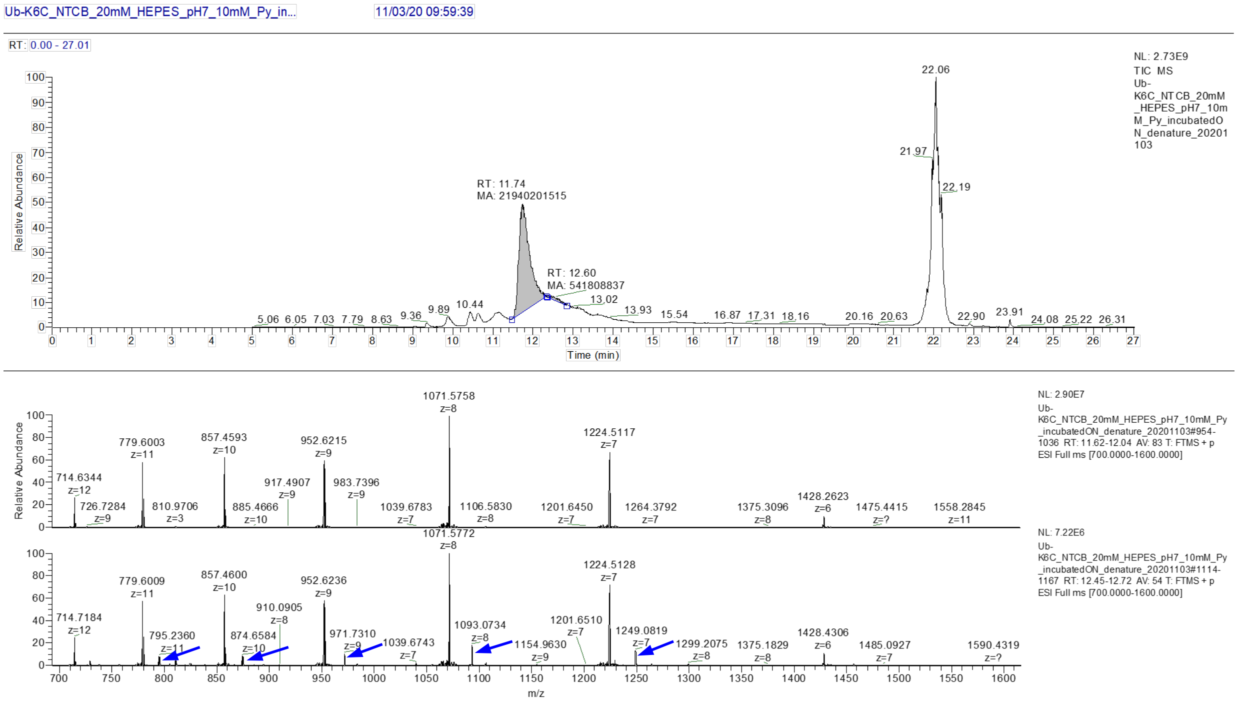

Figure S45. ESI-LC-MS analysis of product mixture of NTCB induced Dha formation reaction using Ub-K6C as the substrate. Reaction condition: 20 mM HEPES pH 7, 6 M GndCl, 37 °C, 18 h incubation. The mass signal pattern in the top mass diagram that came from the 11.74 min peak represented +CN product, Ub-K6C(CN). The mass signal pattern in the bottom mass diagram indicated in blue that came from the 12.60 min peak showed the formation of +TNB product, Ub-K6C(TNB).

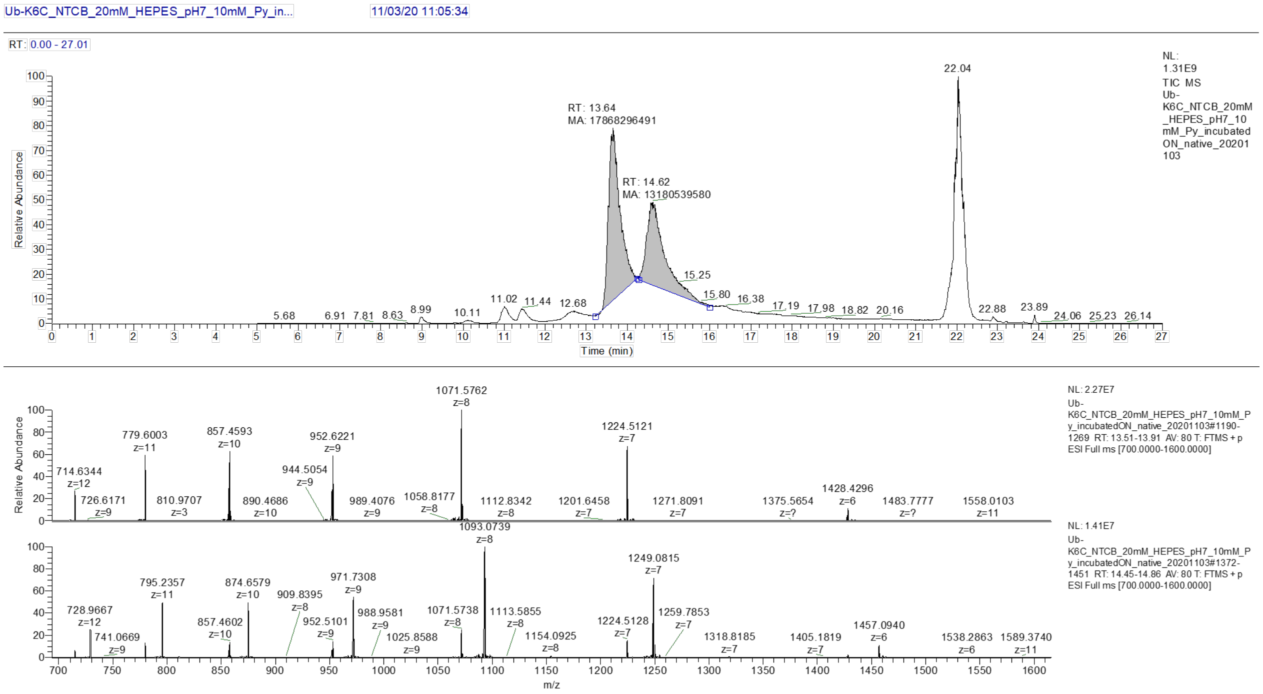

Figure S46. ESI-LC-MS analysis of product mixture of NTCB induced Dha formation reaction using Ub-K6C as the substrate. Reaction condition: 20 mM HEPES pH 7, 37 °C, 18 h incubation. The mass signal pattern in the top mass diagram that came from the 13.64 min peak represented +CN product, Ub-K6C(CN). The mass signal pattern in the bottom mass diagram that came from the 14.62 min peak showed the formation of +TNB product, Ub-K6C(TNB).

Table S1. Sequences of all expressed recombinant proteins

**Ub-G76C-6H**	MQIFVKTLTGKTITLEVEPSDTIENVKAKIQDKEGIPPDQQR LIFAGKQLEDGRTLSDYNIQKESTLHLVLRLRGCHHHHHH
**Ub-K6C**	MQIFVCTLTGKTITLEVEPSDTIENVKAKIQDKEGIPPDQQR LIFAGKQLEDGRTLSDYNIQKESTLHLVLRLRGG
**Ub-K11C**	MQIFVKTLTGCTITLEVEPSDTIENVKAKIQDKEGIPPDQQR LIFAGKQLEDGRTLSDYNIQKESTLHLVLRLRGG
**Ub-K27C**	MQIFVKTLTGKTITLEVEPSDTIENVCAKIQDKEGIPPDQQR LIFAGKQLEDGRTLSDYNIQKESTLHLVLRLRGG
**Ub-K29C**	MQIFVKTLTGKTITLEVEPSDTIENVKACIQDKEGIPPDQQR LIFAGKQLEDGRTLSDYNIQKESTLHLVLRLRGG
**Ub-K33C**	MQIFVKTLTGKTITLEVEPSDTIENVKAKIQDCEGIPPDQQR LIFAGKQLEDGRTLSDYNIQKESTLHLVLRLRGG
**Ub-K48C**	MQIFVKTLTGKTITLEVEPSDTIENVKAKIQDKEGIPPDQQR LIFAGCQLEDGRTLSDYNIQKESTLHLVLRLRGG
**Ub-K63C**	MQIFVKTLTGKTITLEVEPSDTIENVKAKIQDKEGIPPDQQR LIFAGKQLEDGRTLSDYNIQCESTLHLVLRLRGG
**FLAG-NEDD8-G76C-6H**	MDYKDDDDKAAMLIKVKTLTGKEIEIDIEPTDKVERIKER VEEKEGIPPQQQRLIYSGKQMNDEKTAADYKILGGSVLHL VLALRGCHHHHHH
**FLAG-MNSFβ-C57S-G74C-6H**	MDYKDDDDKAAMQLFVRAQELHTLEVTGQETVAQIKDH VASLEGIAPEDQVVLLAGSPLEDEATLGQSGVEALTTLEV AGRMLGCHHHHHH
**FLAG-GABARAP-G116C-6H**	MDYKDDDDKAAMKFVYKEEHPFEKRRSEGEKIRKKYPD RVPVIVEKAPKARIGDLDKKKYLVPSDLTVGQFYFLIRKRI HLRAEDALFFFVNNVIPPTSATMGQLYQEHHEEDFFLYIA YSDESVYCHHHHHH
**FLAG-GABARAPL2-G116C-6H**	MDYKDDDDKAAMKWMFKEDHSLEHRSVESAKIRAKYPD RVPVIVEKVSGSQIVDIDKRKYLVPSDITVAQFMWIIRKRIQ LPSEKAIFLFVDKTVPQSSLTMGQLYEKEKDEDGFLYVAY SGENTFCHHHHHH
**FLAG-UFM1-G83C-6H**	MDYKDDDDKAAMSKVSFKITLTSDPRLPYKVLSVPESTPF TAVLKFAAEEFKVPAATSAIITNDGIGINPAQTAGNVFLKH GSELRIIPRDRVCHHHHHH
**FLAG-URM1-G101C-6H**	MDYKDDDDKAAMAAPLSVEVEFGGGAELLFDGIKKHRV TLPGQEEPWDIRNLLIWIKKNLLKERPELFIQGDSVRPGILV LINDADWELLGELDYQLQDQDSVLFISTLHGCHHHHHH
**FLAG-ISG15-C89S-G157C-6H**	MDYKDDDDKAAMGWDLTVKMLAGNEFQVSLSSSMSVS ELKAQITQKIGVHAFQQRLAVHPSGVALQDRVPLASQGLG PGSTVLLVVDKSDEPLSILVRNNKGRSSTYEVRLTQTVAH LKQQVSGLEGVQDDLFWLTFEGKPLEDQLPLGEYGLKPLS TVFMNLRLRGCHHHHHH
**FLAG-SUMO1-C52A-G97C-6H**	MDYKDDDDKAAMSDQEAKPSTEDLGDKKEGEYIKLKVIG QDSSEIHFKVKMTTHLKKLKESYAQRQGVPMNSLRFLFEG QRIADNHTPKELGMEEEDVIEVYQEQTGCHHHHHH

**FLAG-SUMO2-C48A-G93C-6H**	MDYKDDDDKAAMADEKPKEGVKTENNDHINLKVAGQDGSVVQFKIKRHTPLSKLMKAYAERQGLSMRQIRFRFDGQPINETDTPAQLEMEDEDTIDVFQQQTGCHHHHHH
**FLAG-SUMO3-C47A-G92C-6H**	MDYKDDDDKAAMSEEKPKEGVKTENDHINLKVAGQDGSVVQFKIKRHTPLSKLMKAYAERQGLSMRQIRFRFDGQPINETDTPAQLEMEDEDTIDVFQQQTGCHHHHHH
**FLAG-SUMO4-C48A-G93C-6H**	MDYKDDDDKAAMANEKPTEEVKTENNNHINLKVAGQDGSVVQFKIKRQTPLSKLMKAYAEPRGLSMKQIRFRFGGQPISGTDKPAQLEMEDEDTIDVFQQPTGCHHHHHH

Table S2. DNA sequences of all recombinant protein genes

**Ub-G76C-6H**	ATGCAGATCTTCGTGAAGACTCTGACTGGTAAGACCAT<br>CACTCTCGAAGTGGAGCCGAGTGACACCATTGAGAATG<br>TCAAGGCAAAGATCCAAGACAAGGAAGGCATCCCTCCT<br>GACCAGCAGAGGTTGATCTTTGCTGGGAAACAGCTGGA<br>AGATGGACGCACCCTGTCTGACTACAACATCCAGAAAG<br>AGTCCACCCTGCACCTGGTACTCCGTCTCAGAGGTTGCC<br>ATCACCATCACCATCAC
**Ub-K6C**	ATGCAAATATTCGTGTGCACCCTAACTGGTAAGACCAT<br>CACTCTCGAAGTGGAGCCGAGTGACACCATTGAGAATG<br>TCAAGGCAAAGATCCAAGACAAGGAAGGCATCCCTCCT<br>GACCAGCAGAGGTTGATCTTTGCTGGGAAACAGCTGGA<br>AGATGGACGCACCCTGTCTGACTACAACATCCAGAAAG<br>AGTCCACCCTGCACCTGGTACTCCGTCTCAGAGGTGGT
**Ub-K11C**	ATGCAGATCTTCGTGAAAACCCTAACTGGTTGCACCATC<br>ACTCTCGAAGTGGAGCCGAGTGACACCATTGAGAATGT<br>CAAGGCAAAGATCCAAGACAAGGAAGGCATCCCTCCTG<br>ACCAGCAGAGGTTGATCTTTGCTGGGAAACAGCTGGAA<br>GATGGACGCACCCTGTCTGACTACAACATCCAGAAAGA<br>GTCCACCCTGCACCTGGTACTCCGTCTCAGAGGTGGT
**Ub-K27C**	ATGCAGATCTTCGTGAAGACTCTGACTGGTAAGACCAT<br>CACTCTCGAAGTGGAGCCGAGTGACACCATTGAGAATG<br>TCTGCGCAAAGATCCAAGACAAGGAAGGCATCCCTCCT<br>GACCAGCAGAGGTTGATCTTTGCTGGGAAACAGCTGGA<br>AGATGGACGCACCCTGTCTGACTACAACATCCAGAAAG<br>AGTCCACCCTGCACCTGGTACTCCGTCTCAGAGGTGGT
**Ub-K29C**	ATGCAGATCTTCGTGAAGACTCTGACTGGTAAGACCAT<br>CACTCTCGAAGTGGAGCCGAGTGACACCATTGAGAATG<br>TCAAGGCATGCATCCAAGACAAGGAAGGCATCCCTCCT<br>GACCAGCAGAGGTTGATCTTTGCTGGGAAACAGCTGGA<br>AGATGGACGCACCCTGTCTGACTACAACATCCAGAAAG<br>AGTCCACCCTGCACCTGGTACTCCGTCTCAGAGGTGGT
**Ub-K33C**	ATGCAGATCTTCGTGAAGACTCTGACTGGTAAGACCAT<br>CACTCTCGAAGTGGAGCCGAGTGACACCATTGAGAATG<br>TCAAGGCAAAGATCCAAGACTGCGAAGGCATCCCTCCT<br>GACCAGCAGAGGTTGATCTTTGCTGGGAAACAGCTGGA<br>AGATGGACGCACCCTGTCTGACTACAACATCCAGAAAG<br>AGTCCACCCTGCACCTGGTACTCCGTCTCAGAGGTGGT
**Ub-K48C**	ATGCAGATCTTCGTGAAGACTCTGACTGGTAAGACCAT<br>CACTCTCGAAGTGGAGCCGAGTGACACCATTGAGAATG<br>TCAAGGCAAAGATCCAAGACAAGGAAGGCATCCCTCCT<br>GACCAGCAGAGGTTGATCTTTGCTGGGTGCCAGCTGGA<br>AGATGGACGCACCCTGTCTGACTACAACATCCAGAAAG<br>AGTCCACCCTGCACCTGGTACTCCGTCTCAGAGGTGGT
**Ub-K63C**	ATGCAGATCTTCGTGAAGACTCTGACTGGTAAGACCAT<br>CACTCTCGAAGTGGAGCCGAGTGACACCATTGAGAATG

	TCAAGGCAAAGATCCAAGACAAGGAAGGCATCCCTCCTGACCAGCAGAGGTTGATCTTTGCTGGGAAACAGCTGGAAGATGGACGCACCCTGTCTGACTACAACATCCAGTGCGAGTCCACCCTGCACTTGGTACTCCGTCTCAGAGGTGGT
**FLAG-NEDD8-G76C-6H**	ATGGACTACAAAGACGATGATGACAAAGCTGCAATGCTGATCAAGGTGAAGACTCTGACGGGTAAAGAGATTGAAATTGACATTGAGCCAACCGACAAAGTTGAACGCATCAAAGAGCGCGTCGAGGAGAAGGAGGGTATCCCACCACAGCAACAACGTCTGATCTATAGCGGTAAGCAAATGAACGACGAGAAGACGGCAGCGGACTACAAGATCCTGGGTGGCAGCGTTCTGCATCTGGTGCTGGCTCTGCGTGGCTGCCATCATCACCACCATCAC
**FLAG-MNSFβ-C57S-G74C-6H**	ATGGACTACAAAGACGATGATGACAAAGCTGCAATGCAACTGTTCGTTCGTGCACAGGAGCTGCACACCCTGGAAGTGACTGGCCAAGAAACGGTGGCACAGATCAAAGACCACGTGGCGAGCCTGGAGGGTATTGCGCCAGAGGATCAAGTCGTCCTGCTGGCAGGTAGCCCACTGGAAGACGAGGCAACGCTGGGCCAGAGCGGTGTGGAGGCTCTGACGACGCTGGAGGTTGCCGGTCGTATGCTGGGCTGCCATCATCACCACCATCAC
**FLAG-GABARAP-G116C-6H**	ATGGACTACAAAGACGATGATGACAAAGCTGCAATGAAATTCGTGTACAAGGAGGAGCACCCATTTGAGAAACGCCGTAGCGAGGGTGAGAAGATCCGTAAGAAGTATCCAGATCGTGTGCCAGTGATCGTCGAGAAAGCTCCGAAAGCTCGCATTGGTGACCTGGACAAGAAGAAATACCTGGTTCCAAGCGATCTGACGGTTGGCCAGTTCTACTTCCTGATTCGTAAACGCATCCACCTGCGTGCCGAGGATGCCCTGTTCTTCTTCGTCAATAACGTTATTCCACCGACCAGCGCGACGATGGGCCAACTGTATCAAGAACATCACGAAGAGGATTTCTTTCTGTATATCGCATACAGCGACGAAAGCGTGTATTGCCATCATCACCACCATCAC
**FLAG-GABARAPL2-G116C-6H**	TATGGACTACAAAGACGATGATGACAAAGCTGCAATGAAGTGGATGTTTAAGGAGGACCATAGCCTGGAACATCGCAGCGTGGAGAGCGCGAAGATCCGTGCGAAGTATCCGGATCGTGTGCCAGTGATTGTGGAGAAAGTCAGCGGCAGCCAGATCGTCGATATTGACAAACGTAAGTATCTGGTCCCAAGCGACATTACCGTCGCGCAGTTTATGTGGATCATTCGTAAACGTATCCAGCTGCCAAGCGAGAAGGCGATCTTTCTGTTCGTCGATAAGACCGTGCCGCAAAGCAGCCTGACGATGGGCCAACTGTACGAGAAGGAGAAGGATGAAGATGGTTTCCTGTACGTTGCTTACAGCGGTGAGAACACGTTCTGCCATCATCACCACCATCAC
**FLAG-UFM1-G83C-6H**	ATGGACTACAAAGACGATGATGACAAAGCTGCAATGAGCAAGGTCAGCTTCAAGATTACGCTGACTAGCGATCCGCGTCTGCCGTACAAGGTGCTGAGCGTTCCAGAGAGCACGCCGTTCACCGCTGTCCTGAAGTTTGCGGCAGAAGAGTTC

	AAAGTTCCGGCTGCTACCAGCGCCATTATCACTAACGA CGGCATTGGCATTAACCCAGCCCAAACTGCCGGTAATG TGTTCCTGAAACACGGTAGCGAGCTGCGCATCATCCCA CGTGATCGTGTCTGCCATCATCACCACCATCAC
**FLAG-URM1-G101C-6H**	ATGGACTACAAAGACGATGATGACAAAGCTGCAATGGC AGCTCCGCTGAGCGTGGAAGTTGAATTTGGCGGTGGTG CGGAGCTGCTGTTTGACGGCATTAAGAAACACCGCGTT ACCCTGCCAGGTCAGGAAGAGCCGTGGGACATTCGTAA CCTGCTGATTTGGATCAAGAAGAACCTGCTGAAAGAAC GCCCAGAACTGTTCATTCAGGGTGATAGCGTTCGCCCA GGTATCCTGGTTCTGATTAACGATGCGGACTGGGAACT GCTGGGTGAACTGGATTACCAACTGCAAGATCAGGACA GCGTTCTGTTCATTAGCACCCTGCATGGTTGTCATCATC ACCACCATCAC
**FLAG-ISG15-C89S-G157C-6H**	ATGGACTACAAAGACGATGATGACAAAGCTGCAATGGG CTGGGACCTGACTGTCAAGATGCTGGCAGGTAACGAGT TTCAGGTTAGCCTGAGCAGCAGCATGAGCGTGAGCGAG CTGAAAGCTCAGATTACTCAGAAGATTGGTGTCCACGC CTTTCAGCAACGTCTGGCAGTGCACCCGAGCGGTGTGG CTCTGCAAGACCGTGTCCCACTGGCCAGCCAAGGTCTG GGTCCGGGTAGCACCGTCCTGCTGGTGGTGGACAAGAG CGACGAACCGCTGAGCATTCTGGTGCGTAACAACAAAG GTCGCAGCAGCACGTACGAGGTTCGCCTGACGCAAACT GTGGCTCACCTGAAACAACAGGTGAGCGGTCTGGAGGG TGTGCAAGACGATCTGTTCTGGCTGACGTTTGAGGGTA AACCACTGGAAGACCAACTGCCGCTGGGTGAATATGGC CTGAAGCCGCTGAGCACTGTGTTCATGAACCTGCGCCT GCGTGGTTGCCATCATCACCACCATCAC
**FLAG-SUMO1-C52A-G97C-6H**	ATGGACTACAAAGACGATGATGACAAAGCTGCAATGAG CGACCAGGAGGCAAAGCCAAGCACTGAAGACCTGGGC GACAAGAAGGAGGGTGAGTATATCAAACTGAAGGTCAT TGGCCAAGATAGCAGCGAGATCCACTTCAAAGTGAAGA TGACTACCCATCTGAAGAAGCTGAAAGAAAGCTACGCG CAACGTCAGGGCGTGCCAATGAATAGCCTGCGTTTCCT GTTTGAGGGTCAACGCATCGCAGATAATCATACGCCGA AGGAGCTGGGCATGGAAGAGGAGGATGTCATTGAGGTC TACCAAGAACAAACGGGCTGTCATCATCACCACCATCA C
**FLAG-SUMO2-C48A-G93C-6H**	ATGGACTACAAAGACGATGATGACAAAGCTGCAATGGC GGACGAGAAGCCGAAAGAAGGCGTCAAGACTGAGAAC AATGATCACATTAACCTGAAAGTTGCTGGTCAGGACGG TAGCGTCGTGCAGTTTAAGATCAAACGCCATACTCCGCT GAGCAAGCTGATGAAGGCCTACGCGGAACGCCAAGGTC TGAGCATGCGTCAAATTCGTTTCCGCTTTGATGGCCAAC CAATCAATGAAACCGATACTCCAGCGCAGCTGGAAATG

	GAAGATGAGGATACTATCGATGTCTTCCAGCAACAGAC GGGCTGCCATCATCACCACCATCAC
**FLAG-SUMO3-C47A-G92C-6H**	ATGGACTACAAAGACGATGATGACAAAGCTGCAATGTC TGAGGAGAAGCCGAAAGAAGGCGTCAAGACTGAGAAC GATCACATTAACCTGAAAGTTGCTGGTCAGGACGGTAG CGTCGTGCAGTTTAAGATCAAACGCCATACTCCGCTGA GCAAGCTGATGAAGGCCTACGCGGAACGCCAAGGTCTG AGCATGCGTCAAATTCGTTTCCGCTTTGATGGCCAACCA ATCAATGAAACCGATACTCCAGCGCAGCTGGAAATGGA AGATGAGGATACTATCGATGTCTTCCAGCAACAGACGG GCTGCCATCATCACCACCATCAC
**FLAG-SUMO4-C48A-G93C-6H**	ATGGACTACAAAGACGATGATGACAAAGCTGCAATGGC GAATGAGAAACCGACGGAGGAGGTGAAGACGGAGAAC AACAATCACATCAATCTGAAGGTTGCAGGCCAAGATGG TAGCGTGGTGCAGTTCAAGATCAAGCGCCAGACTCCGC TGAGCAAACTGATGAAAGCCTATGCGGAACCACGCGGT CTGAGCATGAAGCAGATTCGCTTCCGCTTTGGTGGCCA GCCAATTAGCGGTACGGACAAACCAGCCCAGCTGGAAA TGGAGGACGAGGATACCATCGATGTGTTCCAGCAACCG ACTGGTTGCCATCATCACCACCATCAC
